# Missing cell types in single-cell references impact deconvolution of bulk data but are detectable

**DOI:** 10.1186/s13059-025-03506-9

**Published:** 2025-04-07

**Authors:** Adriana Ivich, Natalie R. Davidson, Laurie Grieshober, Weishan Li, Stephanie C. Hicks, Jennifer A. Doherty, Casey S. Greene

**Affiliations:** 1 Department of Biomedical Informatics, University of Colorado Anschutz Medical Campus, Aurora, CO USA; 2 Huntsman Cancer Institute, University of Utah, Salt Lake City, UT USA; 3 Department of Biostatistics, Johns Hopkins Bloomberg School of Public Health, Baltimore, MD USA; 4 Department of Biomedical Engineering, Johns Hopkins University, Baltimore, MD USA; 5 Center for Computational Biology, Johns Hopkins University, Baltimore, MD USA; 6 Malone Center for Engineering in Healthcare, Johns Hopkins University, Baltimore, MD USA

## Abstract

**Background:**

Advancements in RNA sequencing have expanded our ability to study gene expression profiles of biological samples in bulk tissue and single cells. Deconvolution of bulk data with single-cell references provides the ability to study relative cell-type proportions, but most methods assume a reference is present for every cell type in bulk data. This is not true in all circumstances—cell types can be missing in single-cell profiles for many reasons. In this study, we examine the impact of missing cell types on deconvolution methods.

**Results:**

Using paired single-cell and single-nucleus data, we simulate realistic scenarios where cell types are missing since single-nucleus RNA sequencing is able to capture cell types that would otherwise be missing in a single-cell counterpart. Single-nucleus sequencing captures cell types absent in single-cell counterparts, allowing us to study their effects on deconvolution. We evaluate three different methods and find that performance is influenced by both the number and similarity of missing cell types. Additionally, missing cell-type profiles can be recovered from residuals using a simple non-negative matrix factorization strategy. We also analyzed real bulk data of cancerous and non-cancerous samples. We observe results consistent with simulation, namely that expression patterns from cell types likely to be missing appear present in residuals.

**Conclusions:**

We expect our results to provide a starting point for those developing new deconvolution methods and help improve their to better account for the presence of missing cell types. Our results suggest that deconvolution methods should consider the possibility of missing cell types.

**Supplementary Information:**

The online version contains supplementary material available at 10.1186/s13059-025-03506-9.

## Background

Gene expression analysis, particularly through RNA sequencing (RNA-seq), provides a means to characterize transcript levels in complex tissues. Bulk RNA-seq has been widely applied for capturing aggregate snapshots of gene expression within tissues [1, 2]. The advent of single-cell level technologies has brought in a new era, revealing cellular heterogeneity at a higher resolution that was not observable by bulk approaches [3–5].

Previous studies have shown differences in both gene expression and cell proportions between single-cell, single-nucleus, and bulk RNA-seq data [4, 6–10]. In some cases, cell dissociation and processing in single-cell RNA-seq could introduce complete or partial cell loss in cell types that are adherent, sensitive, too large, or have low capture efficiency [6–8]. We expected the impact of this loss might be particularly pronounced when attempting to link observations from single-cell and bulk datasets, since certain cell types may be absent from single-cell profiling.

While not directly observable, cellular heterogeneity could be inferred from bulk samples by bulk deconvolution. Deconvolution methods play a pivotal role in unraveling the cell type proportions within the vast amounts of archival and relatively accessible bulk RNA-seq datasets. Estimating cell-type proportions in bulk datasets is key in identifying cell proportion differences and performing accurate differential gene expression analysis at the cell-type level. These deconvolution methods (when reference-based) rely on a single-cell expression reference (referred to as cell reference) to estimate cell-type proportions in bulk [11, 12]. When single-cell RNA-seq experiments are used as ground truth, the reliability of deconvolution is contingent on the completeness of the reference, posing a critical challenge when certain cell types are absent [10].

High-grade serous ovarian cancer (HGSOC) is a fitting instance of the importance of considering absent cell populations when analyzing single-cell versus bulk RNA sequencing data. In HGSOC, key questions exist as to the nature of previously described transcriptomic subtypes [13, 14], and previous studies have raised the possibility that subtypes may be driven by cell-type proportion differences [15, 16]. Additionally, previous work from our group has found an impact of dissociation on adipocyte-related gene sets, revealing lower expression in dissociated bulk RNA-seq samples compared to their undissociated counterparts, suggesting loss of adipocytes during deconvolution [17]. This discrepancy in expression prompts critical questions about the fidelity of deconvolution methods in the face of missing adipocyte information, particularly when such information could hold clinical relevance. The metastatic potential of HGSOC to the omentum [18, 19], which is rich in adipose tissue, underscores the need to discern the implications of missing adipocyte information in deconvolution analyses. Omentally derived samples may contain more adipocytes than samples from other intraperitoneal locations simply because they are derived from a tissue with more adiposity. We hypothesize this would cause omental bulk samples to have higher expression of adipocytes, while the single-cell RNA-seq counterpart would not have these cells. While our work raises this possibility in HGSOC, missing cell types would impact any tissues where certain cell types are difficult to isolate in a single-cell suspension.

Previous studies have explored the effects on deconvolution performance of the remaining cell types after removing one cell type at a time [20, 21] from the cell reference in the context of testing the robustness of deconvolution methods [11, 22, 23]. These studies focused on the effect of a cell type being removed from the cell reference on the redistribution of predicted cell proportion. Furthermore, they also found that similarity in gene expression, or correlation of expression, between the cell types missing from the cell reference and the remaining cell types in the cell reference can affect the predicted proportions [22]. These previous studies have only examined the effect of removing one cell type at a time and have only removed cell types that are typically not missing from single-cell RNA-seq studies. Also, previous studies have not attempted to recover the missing cell-type information.

Building upon this foundation, we aim to examine the effect on deconvolution performance when multiple cell types are missing from a reference single-cell dataset used in bulk deconvolution, and whether the missing cell-type information is recoverable. We used a curated set of immune cell types, the PBMC3k dataset provided by 10 × Genomics [24], and increased the simulation’s physiological relevance by including white adipose tissue and single-cell RNA-seq data with real missing cell types. Simulated bulk data (referred to as pseudobulks) were generated from these single-cell (and single-nucleus) datasets for deconvolution, incorporating random and realistic proportions with and without Gaussian noise (see the “Methods” section for details). We used three deconvolution methods, non-negative least squares (NNLS [25])—a regression method that solves a least-squares problem with the constraint that all solution coefficients must be non-negative, CIBERSORTx [12], and BayesPrism [11] in our experiments, and calculated residuals matrices by subtracting recreated bulks from original pseudobulks. We examined whether evidence of the missing cell types was present in residuals by applying non-negative matrix factorization (NMF), a dimensionality reduction technique that factorizes a non-negative data matrix into two lower-dimensional non-negative matrices, capturing latent structures or patterns. We also examined a dataset of matched HGSOC bulk and single-cell RNA-seq [17], in which we expect the single-cell data to lack a cell type expected in the bulks (adipocytes). Through these approaches, we carefully examine the consequences of missing cell types on deconvolution outcomes and evaluate the extent to which it is possible to recover absent cell-type information. We do not present a novel method. Instead, our findings suggest paths to improve deconvolution methods, either by adding an iterative step that uses residuals to identify structure that may align with cell types and model it or by using study-specific references alongside large atlas-based panels.

## Results

### Missing cell-type information is present after deconvolution

**Fig. 1:**
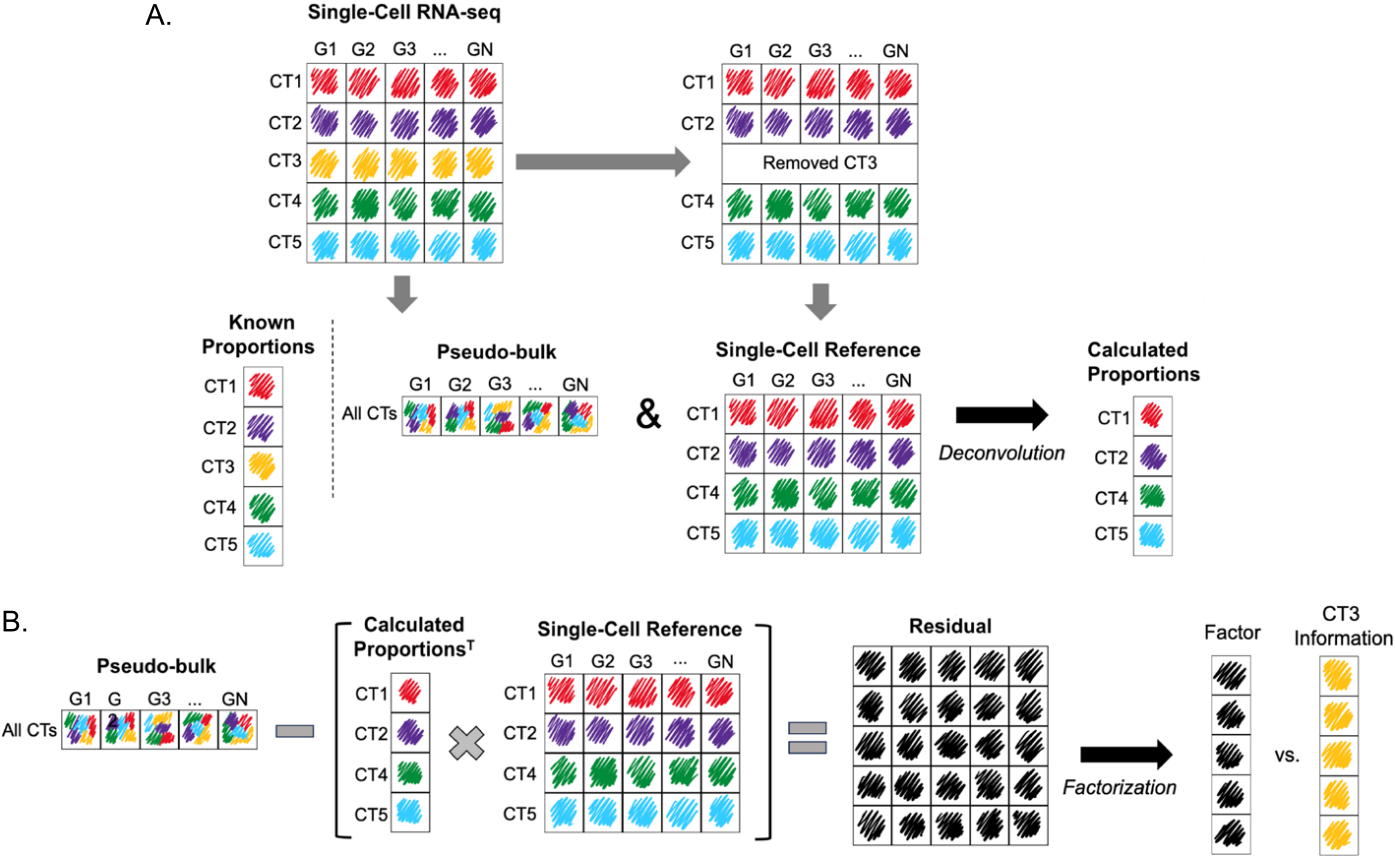
Schematic illustration of experimental design. **A** Single-cell RNA-seq dataset is used to create pseudobulks (bulks with known proportions). Then, a cell type in the single-cell reference is removed. The pseudobulks are deconvolved with that reference, to obtain the calculated proportions. **B** The calculated proportions (a matrix of samples by cell types) are multiplied by the single-cell reference used for deconvolution (a matrix of cell types by genes), to create a bulk-like expression matrix. Then, this bulk-like expression matrix (samples by genes) is subtracted from the original pseudobulks to get the residual matrix, which is factorized to find the missing cell type’s proportions. See Methods: "Residual calculation" section

**Fig. 2:**
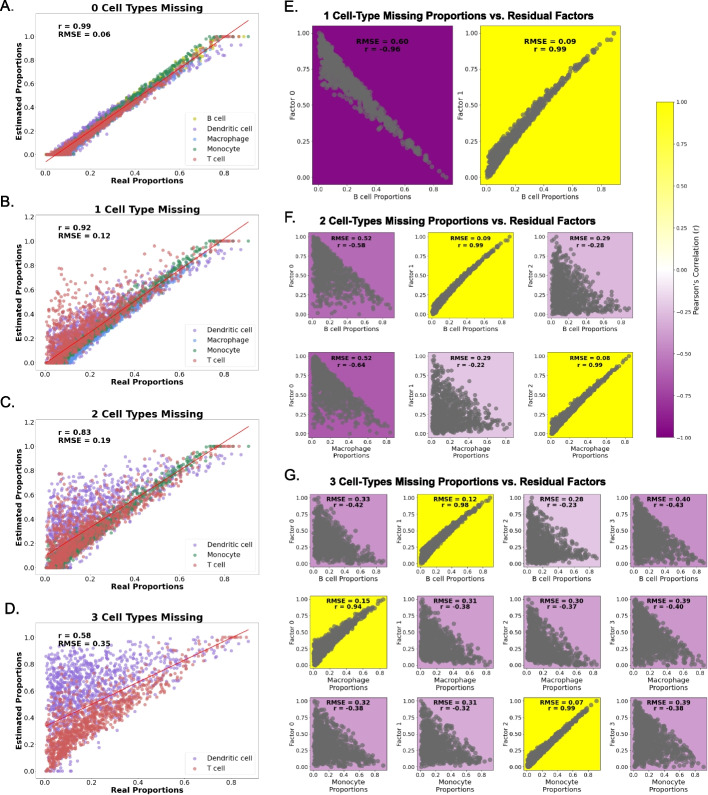
Residual non-negative matrix factorization (NMF) of distinct immune cell types from single-nucleus RNA-seq adipose tissue using non-negative least squares (NNLS). The panels on the left (**A**–**D**) show the deconvolution performance of NNLS in random-proportioned pseudobulks with **A** zero cell types missing, **B** one cell-type missing, **C** two cell types missing, and **D** three cell types missing from the NNLS deconvolution cell reference. Pearson’s correlation (*r*) and root mean square error (RMSE) are noted in each plot between the real and calculated proportions. Panels on the right show each missing cell type’s proportions (real proportions in pseudobulks) correlated with each residual’s NMF factor across the number of missing cell types; **E** reference with one cell type missing, **F** two cell types missing, and **G** three cell types missing. Each row represents one of the missing cell types, and each column represents each normalized NMF factor for each panel. The color represents Pearson's correlation (see color bar), and RMSE is noted in each box

We sought to examine whether methods could recover missing cell-type proportions by first using pseudobulks with 5 distinct immune cell types (Fig. 1A; see the “Methods” section for details). These cell types have variable similarities in their expression profiles, shown in Additional file 1: Fig. S1A. We deconvolved pseudobulks with NNLS [25], the baseline method, with 0, 1, 2, and 3 missing cell types in the cell reference. When no cell types were missing, NNLS demonstrated strong performance, accurately recovering the real proportions of each cell type (Fig. 2A). However, as the number of missing cell types increased, the performance of NNLS gradually declined (Fig. 2B–D). To determine whether the missing cell-type proportions were recoverable, we calculated residuals as shown in Fig. 1B (see the Methods: “Residual calculation” section). When plotting the factorized residuals against the true proportion for each of cell types present in the pseudobulks, NMF factors were found to be correlated with the proportions of each missing cell type (Fig. 2 E, F, and G for 1, 2, and 3 missing cell types, respectively). The missing cell-type proportions show high Pearson’s correlation and low RMSE values for each of the missing cell types (Fig. 2E–G).

### Missing cell-type profiles are present in residuals from widely used methods

NNLS is a straightforward method for this problem, but multiple more sophisticated methods are more commonly deployed in practice. We designed a similar experiment to test two more widely used methods, BayesPrism and CIBERSORTx (which is marker gene-based), alongside NNLS. Additionally, we added more biological complexity to our methodology by creating a new set of pseudobulks from the PBMC3k dataset, which contains cell types with a more similar expression to our previous section (see the “Methods” section for details). This experiment represents a more challenging scenario for deconvolution algorithms.

**Fig. 3:**
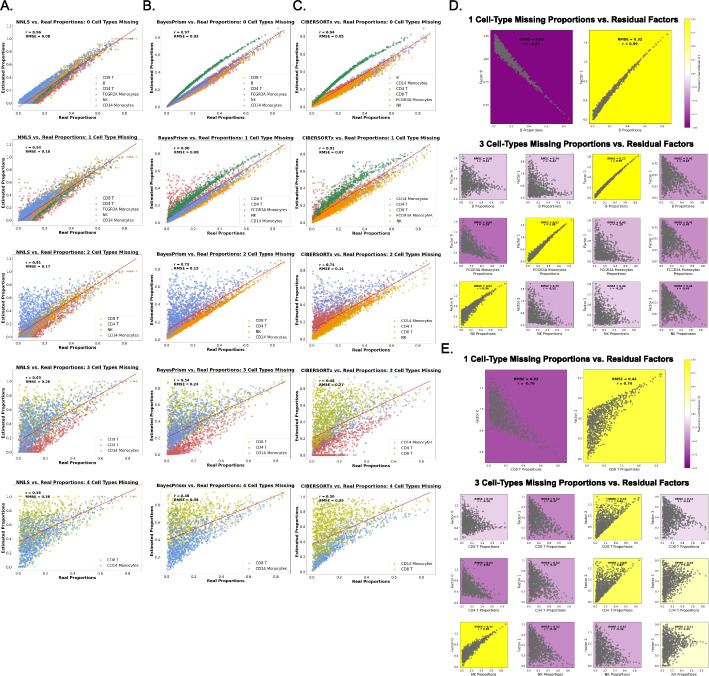
Deconvolution performance across deconvolution methods as we increase the number of missing cell types from the reference (random proportions), and the accuracy of recovering the proportions is dependent on how similar the missing cell types are to the other missing cells in random-proportioned pseudobulks (PBMC3k) deconvolved with Non-Negative Least Squares (NNLS), with residual factorization through non-negative Matrix Factorization (NMF). **A** NNLS, **B** BayesPrism, and **C** CIBERSORTx performance (real vs. estimated) proportions. The first row corresponds to the control (zero missing cell types), the second row corresponds to one missing cell type (B cells), then two missing cell types (B cells, FCGR3A monocytes), three (B cells, FCGR3A monocytes, NK) up to four missing cell types (B cells, FCGR3A monocytes, NK, CD4 T cells) in the last row. Each cell’s proportions are colored a different color (legend), and each plot has Pearson’s correlation (*r*) and root mean square error (RMSE) value noted. The panels on the right (D and E) show the correlation between the residual factor and each missing cell type (real proportions from pseudobulks), comparing the deletion of **D** cell types with distinct expressions are deleted from the single-cell reference, and the residual is factorized and **E** cell types with similar expression are deleted from the single-cell reference, and the residual is factorized

Specifically, we first removed cell types from the deconvolution cell reference for which the reference profiles were relatively uncorrelated (Additional file 1: Fig. S2B, removed cell types are B cells, FCGR3A Monocytes, and NK T cell, and CD4 T, cumulatively). We analyzed these data as in our first experiment, using BayesPrism and CIBERSORTx in addition to NNLS. For BayesPrism and CIBERSORTx, we used each model’s directives according to the original publication while creating the references [11, 12]. For NNLS, the cell reference is created as in the previous experiment (see Methods for details). We observed similar effects on the performance of deconvolution methods as we increased the number of missing cell types. Figure 3 shows deconvolution performance for NNLS, BayesPrism, and CIBERSORTx in panels A–C, respectively.

We next determined whether the residuals from these additional methods similarly contained apparent signal from missing cell types. To do this, we calculated and factorized the residuals. We often found factors highly correlated to each of the missing cell types. We observed this pattern irrespective of method (Figure Additional file 1: Fig. S2A, S3A, and S4A show NNLS, BayesPrism, and CIBERSORTx, respectively). This suggested that information present in the residuals was likely to represent cell types, raising the possibility that future methods might be able to tap into this latent structure.

We hypothesized that the recoverability of this information would depend on the similarity of the missing cell type’s expression profiles, both with other removed cell types and cell types in the pseudobulks, as seen in previous work [22]. We designed an experiment to examine whether similarity influenced recoverability by removing CD8 T cell, CD4 T cell, NK T cell, and FCGR3A monocyte cells, cumulatively, from the same data. These cells have relatively more correlated expression with other cell types (Additional file 1: Fig. S1B) compared to the previously removed cells. We found differences between these conditions (Fig. 3D vs. E). Although deconvolution performance was comparable for all deconvolution methods regardless of whether the missing cell types were similar or not (Additional file 1: Fig. S5: A vs. B, and Fig. S3 A, B, C vs. S6), the recoverability of missing cell types’ proportions appeared substantially more dependent on the identity of the missing cell type. When we remove very distinct cell types, we observe that the true proportion of the cell type uniquely matches to one NMF factor (Fig. 3D). This contrasts with the NMF factors of residuals after more similar cell types are omitted (Fig. 3E).

### Matched single-cell and single-nucleus data enable testing with realistic proportions

**Fig. 4:**
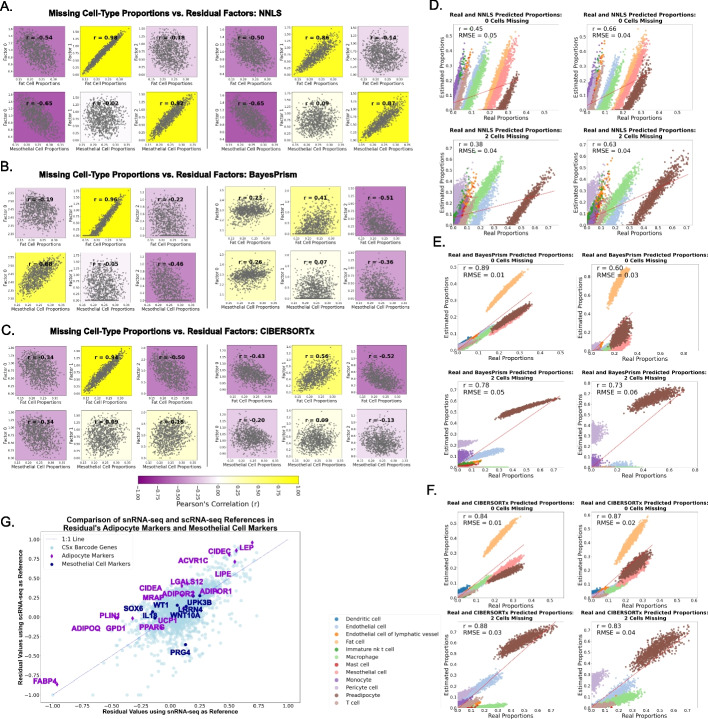
Single-nucleus (snRNA-seq) and single-cell (scRNA-seq)-informed-pseudobulks comparing simulated missing cell types, and real adipose bulks deconvolved with these as reference. For our simulation experiments, pseudobulks were created from snRNA-seq adipose tissue cell expression with realistic proportions as observed in the snRNA-seq data and deconvolved with either scRNA-seq (2 cells missing) or snRNA-seq (no cells missing). See Supplemental Fig. 7C for details on expression and references used for this simulation. The left panels (**A**–**C**) show the correlation between the residual’s NMF factors and each of the missing cell types’ proportions across pseudobulks for **A** non-negative least squares (NNLS),** B** BayesPrism, and **C** CIBERSORTx. Plots in the left column represent pseudobulks made with no noise added, and those in the right column represent pseudobulks with noise added. Pearson’s correlation (*r*) is noted in each plot. Panels on the right (**D**–**F**) show real vs. calculated proportions for pseudobulks of realistic proportions in each of the deconvolution methods. The left column represents pseudobulks with no noise added, and the right column represents bulks with noise added. **D** NNLS, **E** BayesPrism, and** F** CIBERSORTx deconvolution were used. The top plot of each panel represents the deconvolution with no cells missing (same cells as present in pseudobulks), and the bottom plot represents the proportions with two cells missing (no adipocytes or mesothelial cells). The red line in each plot represents the regression fit line. Each plot has root mean square error (RMSE), and r is noted. **G** We deconvolved 43 real bulk adipose tissue samples, and calculated each residual using both the snRNA-seq (*x*-axis) and scRNA-seq (*y*-axis)—which we hypothesize contains no missing cells compared to bulk. The mean is calculated for each gene across samples for both residuals, and these values are compared in a scatterplot for adipocyte and mesothelial cell markers, along with CIBERSORTx barcode genes

Our experiments thus far have constructed scenarios in which the missing cell type is removed without regard to what is physiologically likely or realistic. To examine the effect in a more realistic scenario, we sought to identify a condition where we could create profiles that mimic actual bulk/single-cell discrepancies while still retaining a gold standard for comparison. Publicly available datasets with both single-cell and single-nucleus data that contain adipocytes provided an ideal test case. While mesothelial cells seem to be present in some single-cell sequencing datasets [26, 27], in these datasets we used both adipocytes and mesothelial cells are shown to be lost during single-cell sequencing but the nuclei are preserved and profiled in single-nucleus sequencing [28]. In our experiment, the cell-type proportion in pseudobulks was defined based on cell types observed in single-cell data, while the expression profiles themselves were derived from single-nucleus counterparts. Additional file 1: Fig. S7C shows the data origin for each component of the simulated data. This provided gold standard cell-type proportions with realistic missingness. The single-cell references are based on the single-cell profiles as well, which in this experiment has substantial depletion of two cell types, adipocytes, and mesothelial cells (see Methods for details; Additional file 1: Fig. S7 A–B for cells and proportions present in each dataset). We then examined scenarios with and without added sources of noise. As in previous experiments, the residual is calculated with the deconvolution-calculated proportions and the single-cell reference. The residual is then factorized into 3 factors, from which one is expected to be correlated to the adipocyte’s and one to the mesothelial cell’s proportions. We observed variable results for deconvolution performance between scenarios where pseudobulks had noise added and those without as well as between experiments with random proportions and realistic proportions. Performance was overall lower in pseudobulks of realistic proportions when compared with those created using uniform random proportions for all deconvolution methods (Fig. 4D–F vs. Additional file 1: Fig. S8 D–F). It is important to note that the two missing cell types encompassed over 50% of all cells in the mixtures (Additional file 1: Fig. S8B). When comparing bulks with and without noise added, we also observed an expected decrease in deconvolution accuracy, as seen in Fig. 4D–F (left) vs. Additional file 1: Fig. S8 D–F (left).

We then examined whether the residuals still contained some information on the missing cell types and found that pseudobulks with realistic proportions had a lower correlation of missing cell-type proportions and residual factors when compared to random-proportioned pseudobulks (Fig. 4E–G vs. Additional file 1: Fig. S8 D–E). However, we observed some correlation, especially in NNLS deconvolution (Fig. 4E). When comparing pseudobulks with and without added noise, the noise lowered correlation further across deconvolution methods. Overall, this is not surprising given that implementing NMF in this manner assumes the residual contains no or minimal noise. In the presence of noise, NMF is expected to interpret the noise as part of the underlying factors, leading to inaccurate separation. When deconvolving noisy pseudobulks, BayesPrism showed the lowest correlation (Fig. 4D–F left vs. right, and Additional file 1: Fig. S8 D–F right vs. left) and highest RMSE values (Additional file 1: Fig. S10) in the factor-proportion comparison. This is attributed to BayesPrism’s lower deconvolution accuracy with noise added.

We next sought to determine whether results consistent with this model would be observed in real-world scenarios. We downloaded and processed 43 bulk RNA-seq samples from adipose tissue created as part of the same study from which the single-cell and single-nucleus adipose datasets originate. We performed an analysis reflecting a typical research setting where researchers have a cohort of bulk RNA-seq data that they deconvolve with a single-cell reference dataset from unmatched participants. In this case, we used both single-cell and single-nucleus RNA-seq to serve as references for deconvolution. The single-nucleus RNA-seq dataset has 2 more cell types (mesothelial cells and adipocytes) that were absent from the single-cell profiles. We deconvolve the bulk samples using NNLS, using the single-cell and the single-nucleus separately as references. We then calculate the residual matrix as we have done in the simulation studies. When comparing the residual values of marker genes from adipocytes and mesothelial cells, we observed these marker genes were overall higher in residuals of bulks deconvolved with scRNA-seq as reference compared to residuals of bulks deconvolved with snRNA-seq (Fig. 4G). This supports a model where cell types observed in scRNA-seq might not represent all cells presenting real bulk datasets, which would be reflected by higher expression values in the residuals of marker genes after deconvolution.

### Recovery of missing cell type’s expression HGSOC samples

We next sought to determine whether results consistent with this model would be observed in data derived from high-grade serous ovarian cancer samples for which bulk and matching single-cell data were available. We analyzed a dataset of 8 matched bulk and single-cell RNA-seq, for which we also have bulk RNA-seq counts of tissue that was dissociated before sequencing and thus lacking cells lost in dissociation (dissociated bulks). For some samples, the location of the sample origin was available (Additional file 1: Fig. S10A). We hypothesized that the Dissociated bulks were missing adipocytes when compared to typical non-dissociated bulks (classic bulks). Our experimental design tested whether deconvolution of dissociated bulk (bulk and single-cell have same cell types) yielded a different residual than residuals of bulks with one extra cell type (adipocytes) when compared to their single-cell counterparts, and whether the missing cell’s expression was recoverable through the residual (Additional file 1: Fig. S10B).

**Fig. 5:**
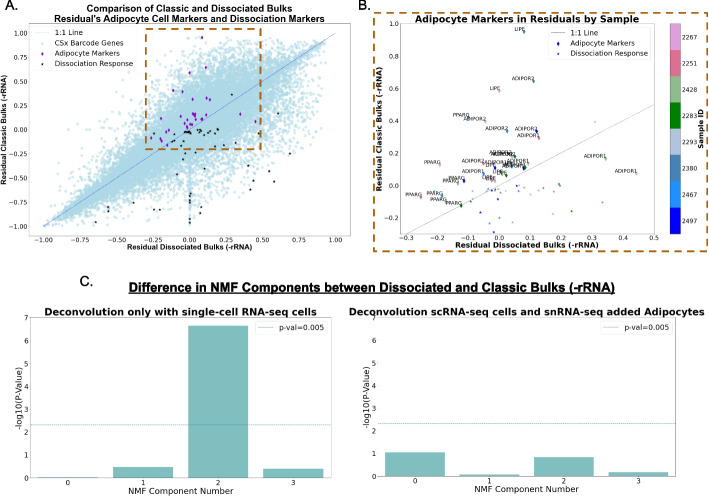
Residual analysis from high grade serous ovarian cancer (HGSOC) dissociated and classic bulk samples deconvolved with NNLS using matched single-cell RNA-seq data.** A**–**B** Comparison of residual values of dissociated bulks (ribosomal RNA depleted (-rRNA)) and classic Bulks (-rRNA) at the gene and sample level. Dissociated bulk residuals are on the *x*-axis, and classic bulks are on the *y*-axis in both panels. CIBERSORTx barcode genes (calculated from all single-cell datasets), adipose markers, and dissociation response genes (from the original paper) are compared. **A** CIBERSORTx barcode genes show no difference between bulks, but adipocyte markers show higher values overall in classic bulks, whereas dissociation response genes have lower values in the residual of dissociated bulks. **B** Zoomed-in version of panel **A** along the dotted line, coloring each gene by which sample it originates from. **C** The difference between in (non-negative matrix factorization) NMF components shows one component, 2, significantly different between the dissociated and classic bulks deconvolved with matched single-cell RNA-seq dataset as reference (left). After deconvolution of the same bulks, but adding an adipose signal to the reference from single-nucleus RNA-seq dataset (right)

We first analyzed whether adipocyte-related genes were overall higher in residuals of classic bulks compared to dissociated bulks. Dissociated genes (characterized in the data’s original publication [24]) were much higher in dissociated bulks. As expected, CIBERSORTx barcode genes showed a roughly even distribution, and adipocyte marker genes were higher in residuals of classic bulks (Fig. 5A–B). We also observed this in violin plots comparing the distributions of Classic and Dissociated residuals by gene group (Additional file 1: Fig. S11).

We then ran NMF on the combined residual, and compared each factor’s weights between dissociates and classic bulks with *t*-tests (see the “Methods” section for details). We found that while most NMF components showed no significant differences between the dissociated and classic bulks (factors 0, 1, and 3, *p*-values > 0.05), factor 2 was significantly different (*p*-values < 0.05), suggesting this component captures a key variation attributable to the dissociation process (Fig. 5C). We hypothesized that the significantly different component between dissociated and classic bulk residuals was related to adipocyte differences. We tested whether including adipocytes in the single-cell RNA reference would remove the significant difference between the residuals. GO analysis did not reveal any obvious cell-type-related processes (Additional file 1: Fig. S12), but we found that adding this cell type to the reference was sufficient to remove the difference between the residuals (Fig. 5C), suggesting adipocyte loss through the dissociation process.

Finally, we considered that GO terms might be better reflected in the orthogonal representation of PCA than the compositional representation of NMF (e.g., because GO process members could positively or negatively regulate the process). We analyzed the residuals of Dissociates and Classic bulks with PCA (principal component analysis) (see Methods for details) (Additional file 1: Fig. S12A). We performed GO analysis on the statistically significantly different components and found biological processes heavily associated with adipocytes (Additional file 1: Fig. S12B).

## Discussion

The goal of this paper is not to present a novel method but to raise awareness about the intricacies of RNA-seq technologies. Our study aimed to explore the deconvolution performance of NNLS, BayesPrism, and CIBERSORTx under varying conditions of missing cell types and realistic proportions using both simulated pseudobulks and experimental bulk RNA-seq datasets. We describe the impact of missing cell types, their similarity, and having bulks with realistic proportions on the deconvolution accuracy.

In our experiments with distinct immune cell types, we observed a decrease in deconvolution performance as the number of missing cell types increased, similarly to previous work done with one cell type missing [11, 20]. We also observed the missing cell types’ proportions have a very high correlation to one of the residual factors.

When applying the deconvolution methods to the PBMC3k dataset, we observed that the cell type’s similarity plays a crucial role in whether we can recover the missing cell types’ proportions through NMF. The more similar the cell type is to others, the more that cell type’s corresponding NMF factor is convolved with others in all three deconvolution methods (Fig. 2). This is in agreement with previous work [22].

Furthermore, we found that deconvolution performance was consistent regardless of the expression similarity among missing cell types. We also found comparable performance across NNLS, BayesPrism, and CIBERSORTx in recovering missing cell types’ proportions. However, the NNLS residual factor had a higher correlation overall, which we attribute to NNLS being a regression model, and our methodology of calculating the residual is based on regression’s residuals. Notably, we tested three deconvolution methods with distinct methodologies and wide use, but our framework for evaluating missing cell types remains applicable to any method, including deep learning approaches, which we do not evaluate in this study.

The impact of realistic proportions and simulated noise on deconvolution performance was explored in the context of adipose tissue datasets. Realistic proportions of adipocytes and mesothelial cells, which collectively constituted a significant portion of the mixtures, led to a decrease in deconvolution accuracy across all methods. The addition of noise further diminished the correlation between the missing cell types’ proportions and residual factors. Despite this, the residuals still contained information about the missing cell types, emphasizing the potential utility of our approach even under challenging conditions. Furthermore, when comparing the residual’s values of real adipose tissue bulk RNA-seq dataset deconvolved with snRNA-seq and with scRNA-seq, we observe that the marker genes of the hypothesized missing cell types, adipocytes and mesothelial cells, are overall higher in residuals of real bulks deconvolved with scRNA-seq, consistent with our model.

Applying our approach to data generated to understand HGSOC cell-type proportions suggests that such datasets exhibit signatures of these missing cell types. The results also enable hypothesis generation about samples with incomplete metadata. For example, of the 4 samples of unknown origin, we found one that appeared to have a strong adipose component, which suggests that it may be of omental origin.

We conducted exploratory experiments on NMF to compare the residuals of dissociated and non-dissociated HGSOC bulk RNA-seq samples. This analysis identified differences suggesting potential missing cell types, particularly adipocytes. Incorporating adipocytes, the hypothesized missing cell type, to the deconvolution reference was enough to remove the difference in NMF components from Dissociates and Classic bulks, demonstrating how residual analysis can refine deconvolution accuracy. These results highlight a practical way to improve deconvolution methods by leveraging residual insights and serve as a starting point for future methods seeking to uncover missing cell-type identities within bulk RNA-seq data.

We recognize the value of applying deconvolution methods to bulk RNA-seq data with known ground truth proportions, such as flow cytometry measurements, but obtaining true cell type proportions from bulk data is inherently challenging due to tissue destruction during sequencing. To address this, we used paired single-cell and single-nucleus data to create pseudobulk samples, incorporating Gaussian noise to simulate experimental variability. Additionally, we applied our methods to real bulk RNA-seq data from high-grade serous ovarian cancer (HGSOC) samples, providing further insights into the challenges of deconvolution in real-world settings. While single-cell data from the same samples serve as an approximate ground truth, dissociation biases complicate direct comparisons. Our combined approach of simulations and real-world application thus addresses key challenges in bulk deconvolution without relying on an exact ground truth.

While this study offers the characterization of the “missing-cell problem” in bulk deconvolution, there are important limitations to consider. This study is limited by the assumption that the presence and number of missing cell types are known a priori. This represents a significant challenge for applications involving complex tissue samples where the cell types present are uncertain. We anticipate future research to determine whether or not residuals alone can reveal what is missing. This intersects with another important limitation of this study: the interpretation of residuals, which may reflect missing cell types, technical noise, or both. While residuals provide a promising avenue for detecting missing cell types, distinguishing between biological signals and noise remains a significant challenge. The effectiveness of this approach is likely influenced by the deconvolution method used, further complicating its application. To address this, we emphasize the need for future work to develop robust methodologies and quantitative metrics to characterize the origins of residuals more precisely. Future work should focus on establishing a comprehensive, quantitative framework for detecting, estimating, and characterizing missing cell types, as well as the signals present in the residual, to enhance the robustness of deconvolution methods in real-world scenarios.

We acknowledge the existence of methods like EPIC [29], SECRET [30], and PREDE [31] that address incomplete reference datasets in deconvolution. However, these methods require prior knowledge of missing cell types or assumptions about their distinctness and prevalence, which may not hold in real-world applications where multiple cell types are missing. Our study introduces a new experimental design using single-nucleus RNA-seq data to simulate realistic scenarios where cell types are absent, highlighting the unresolved issue of deconvolution biases in such cases. We emphasize that our contribution lies in evaluating these challenges through a novel framework, rather than proposing a new deconvolution method.

## Conclusions

We examined the impact of missing or lost cell types on deconvolution methods, emphasizing the importance of understanding these constraints for meaningful biological insights. As researchers delve into increasingly complex tissues, these types of factors can influence results and estimates of cell proportions within the tissue. Recognizing these constraints not only refines our interpretation of results but also paves the way for continued advancements in technology and methodologies. The presence of missing cell type’s signal in the residual suggests multiple paths for future methods; while the ideal reference is likely matched from the same participants, perhaps residuals could enable searching of cell-type reference libraries for profiles to augment deconvolution. Alternatively, iterative procedures could be used to estimate missing cell types, refine deconvolution, and potentially repeat the process. In either case, acknowledging and addressing these challenges will undoubtedly enhance the robustness and reliability of our findings, fostering a more profound and accurate understanding of the biological intricacies encoded in RNA-seq data.

## Methods

### Data

We used three datasets for this study, all of which are available online as count matrices through NCBI GEO. Each was processed with adjustments for each data type, outlined in Additional file 2: Table 1, along with cell-type assignments, data links, and related figures. The cell types were assigned according to each data set’s original study, apart from the 10 × Genomics PBMC3k dataset [24], for which we used Scanpy’s [32] tutorial (https://scanpy-tutorials.readthedocs.io/en/latest/pbmc3k.html) cell assignments.

We first used single-nucleus white adipose tissue RNA-seq data and extracted the expression of 5 cell types (macrophage, T cell, B cell, monocyte, dendritic cell). We then expanded in complexity and used a PBMC3k single-cell RNA-seq dataset from 10 × Genomics. We also used the single-cell and single-nucleus dataset of adipose tissue with real missing cell types in the single-cell counterparts compared to the single-cell. Cell-type similarities vary between each dataset, and these are shown in a correlation distance dendrogram in Additional file 1: Fig. S5. We created simulated bulks with each of these datasets.

For real bulk RNA-seq datasets, we leveraged HGSOC datasets from a previous study [17] from our group and the adipose tissue bulk samples used in the same study as the above scRNA-seq and snRNA-seq datasets [28]. The HGSOC data contains data on 8 tumors, with matched single-cell RNA-seq, classic bulk RNA-seq with ribosomal RNA depletion (classic bulk -rRNA), dissociated cells that were bulk RNA-sequenced with ribosomal RNA depletion (dissociated bulk -rRNA) and dissociated cells that were also bulk RNA-sequenced with poly-A tail capture (dissociated-bulk polyA). Our dataset consists of 2 known omental samples, 2 ovarian samples, and 4 samples with unknown origin.

### Pseudobulks for deconvolution of known cell-type proportions

Pseudobulks were generated using single-cell or single-nucleus data from the sources detailed in the “Data” section, apart from the HGSOC data, for which we have matched real bulks. We created pseudobulks with both random proportions and realistic proportions. The proportions for each pseudobulk with random proportions are defined as a vector of random numbers for random proportions, enforcing a sum-to-one constraint. For the realistic proportions, we used a vector of the real cell proportions in the data, with added Gaussian noise for variability in each cell type’s proportions for all bulks.

For each pseudobulk, we defined the total number of cells to be 5000. We multiplied the proportion vector (described above), defined as either random or realistic, by the number of cells per bulk (5000) to get the number of cells, per cell type, in each bulk. Cells were then sampled from the original dataset to match this number, representing the number of cells per bulk. We summed the expression of the sampled cells for each gene, giving the final pseudobulk count. We tested our approach with our initial curated dataset of distinct immune cell types using 10, 20, 100, and 1000 pseudobulks and found consistent results (Additional file 1: Fig. S14). We did all simulations with 1000 pseudobulks in alignment with the deconvolution benchmarking study.

Additionally, to assess the performance with rare cell types, we performed an additional analysis in which we restricted to samples for which at least one missing cell type comprised less than 5% of the sample, and when all missing cell types comprised less than 5% of the sample. Our findings demonstrated that NMF could recover these rare cell-type proportions with high Pearson’s correlation and low RMSE values, even in complex scenarios where at least one of the missing cell types was below 5% (Additional file 1: Fig. S15 A and B). When all missing cells comprise less than 5% of the sample, performance decreases, but some signals are still present (Additional file 1: Fig. S15 A and C).

In the experiments using real missing cell types in adipose tissue, we also have single-nucleus and single-cell data. The pseudobulks were created by using the expression of single-nucleus cells (no cells missing). For the pseudobulks with noise added used in this experiment, we added Gaussian noise to simulate library size and general variability noise seen in real bulk RNA-seq. We end up with four types of pseudobulks (random with no noise, random with noise, realistic with no noise, realistic with noise), all based on single-nucleus cells’ expression.

### Cell reference for deconvolution

The single-cell references were created using the same single-cell dataset as the pseudobulks, containing either the same cell types as the pseudobulks or with missing cell types. The single-cell reference for deconvolution was constructed by sampling with replacement from each cell type to reach a uniform count of 10,000 cells per cell type to standardize the data and reduce potential bias caused by varying cell counts across types. We then added these counts per cell type, which gave us a signature expression per cell type, per gene. We used this reference as having 0 missing cell types, and subsequently iteratively depleted the reference of cumulative randomly chosen (or specified) cell types. We removed cells until the reference contained a minimum of 2 present cell types. The pseudobulks of five distinct cell types from single-nucleus adipose tissue were deconvolved with 0, 1, 2, and 3 missing cell types. The PBMC3k pseudobulks were deconvolved with 0,1, 2, 3, and 4 missing cell types, both with and without similar expression. For the single-cell/single-nucleus adipose tissue, we only considered 0 missing cell types (real single-nucleus proportions) or 2 missing cell types (real single-cell proportions). This is shown in Fig. 5. For each experiment, we end with a distinct single-cell reference matrix with a specific number of known missing cell types compared to the pseudobulks.

For NNLS [25], the single-cell reference and each pseudobulk are kept in the linear scale to improve deconvolution performance, as observed by a benchmarking study [20]. We MinMax scaled the pseudobulks and the reference prior to deconvolution. Prior to MinMax scaling, we clipped the top 95% highest values from the matrix, so that gene expression from high-expressing genes would not skew our scaled values. The pseudobulks were not scaled for BayesPrism and CIBERSORTx, in accordance with the methodology’s specifications. The NNLS reference was filtered to only contain the barcode genes computed through the CIBERSORTx reference matrix with 0 cells missing (containing all cells’ barcode genes) to maximize each cell’s signal. BayesPrism and CIBERSORTx references contained all genes of their respective references as computed by the deconvolution method.

For the adipose single-cell/single-nucleus experiment (Fig. 4 and Additional file 1: Fig. S7), single-nucleus data was used as the reference (with no missing cell types) since it contains all cells and matched the expression of the pseudobulks. For the reference with missing cell types(2 in this real case), we created the reference with the single-cell proportions (missing adipocytes and mesothelial cells) but sampling the expression of each cell from the single-nucleus data. In the case of real HGSOC bulk deconvolution, we combined the single-cell matched datasets from the bulks into one reference used to deconvolve all the bulks to maximize the number of cells present.

The adipose single-nucleus/single-cell dataset contains cells from multiple patients, making it too large for all cells to be reasonably used in deconvolution for CIBERSORTx and BayesPrism which create a single-cell reference based on each cell. BayesPrism literature shows robust performance with references containing between 100 to 500 cells [11], and CIBERSORTx [12] only tested performance up to 500 cells per cell type. Therefore, we limited the number of cells to 2000 for these deconvolution methods when using these large data without compromising performance.

### Deconvolution (NNLS, CIBERSORTx, BayesPrism)

We used NNLS (regression-based), CIBERSORTx (marker-gene based), and BayesPrism (Bayesian-based) deconvolution in both pseudobulks with ground truth and real bulks. Software versions for each deconvolution method used are NNLS v.1.13.0, BayesPrism v2.2, CIBERSORTx v1.05.

For CIBERSORTx and BayesPrism, we followed the specifications of the methodology for deconvolution. All methods were given a reference with no cell types missing and with cell types missing as outlined in cell reference for deconvolution section, and our pseudobulk with all cell types to be deconvolved. This process was repeated for 1000 [20] pseudobulks, with references with no missing cell types and with missing cell types. The NNLS deconvolution was run with SciPy’s NNLS algorithm [25] with default settings. BayesPrism was run through InstaPrism [33], and CIBERSORTx was run through the Docker container with a token from the CIBERSORTx team.

### Residual calculation

Residuals were calculated by subtracting the recreated bulks from the original input pseudobulks matrix, as shown in Fig. 1B. The bulk-like matrix is computed by multiplying the deconvolution-calculated proportions by the single-cell gene-expression reference (MinMax scaled). This single-cell gene expression reference is a matrix of cell types (rows) by genes (columns), which is used as the cell reference for deconvolution. We used BayesPrism and NNLS cell reference when calculating their respective residuals. For CIBERSORTx, this method utilizes a “signature matrix” rather than a gene expression reference matrix. This signature matrix represents relative expression rather than absolute gene expression per cell type, making it unsuitable for reconstructing bulk gene expression directly. To be able to calculate a comparable residual, we used the cell reference as computed for NNLS for the recreated matrix calculation instead. Each single-cell expression matrix is then multiplied with the estimated proportions from each respective method to get the bulk-like matrix (samples by genes).

To get the final residual matrix, we subtract each method’s bulk-like matrix from the original pseudobulks (samples by genes). This residual represents the deviation from the expected proportions based on the deconvolution results.

### Residual factorization and evaluation

The residual matrices contain pseudobulks (rows) by genes (columns) for each reference used in deconvolution (with all cell types and with missing cell types). Because the residuals have negative values, we shift the distribution of values by the minimum value needed for all values to be greater than 0. We factorize each residual using non-negative matrix factorization (NMF) [25] with Nonnegative Double Singular Value Decomposition (nnsvd) initiation, and with maximum iterations of 10,000 for each. We set the number of NMF factors across all analyses to be $\text{number of factors}=\text{number of missing cell types}+1$. The extra component is meant to account for variability in the data from sources other than a missing cell type. Each NMF component’s values are scaled to be between 0 and 1 (the scale of proportions) and further compared to each of the missing cell type’s proportions for only pseudobulks in which we expect the distribution of proportions to be distributed between nearly 0 and 1 (random proportions). For realistic proportions, we do not expect the proportions to have that distribution, and therefore the RMSE is not meaningful and not calculated in those cases. For random proportions, the RMSE and Pearson’s correlation for each combination of component missing cell type is computed and recorded. For realistic proportions, only Pearson’s correlation is recorded. In the current study, we do not evaluate metrics to detect the presence or number of missing cell types a priori, but analysis on these metrics can be found in the GitHub repository under https://github.com/greenelab/pred_missing_celltypes/tree/main/evaluation_experiments/EXP1_detection/ipynb.

### Evaluation of residuals real bulk RNA-seq data

For the adipose tissue bulks, we deconvolve the same 43 real samples with 2 separate references; one with the scRNA-seq cells and one with the snRNA-seq cells of adipose tissue using NNLS. We calculate each residual as outlined above for each deconvolution separately. We then compare genes belonging to the hypothesized missing cells, as seen in snRNA-seq compared to scRNA-seq, adipocyte, and mesothelial cell markers. Genes that have high values in the residuals represent high unexplained expression by the deconvolution method. We then compare residual values of CIBERSORTx, barcode, adipocyte markers, and mesothelial cell markers genes in a scatter plot by plotting the average value per gene across the 43 bulk samples (Fig. 4G). Values along the 1 to 1 line represent genes that are equal in both residuals.

For the HGSOC data analysis, we used the real Classic (-rRNA) or Dissociated (-rRNA and polyA) bulks for deconvolution using NNLS, (experimental design, Additional file 1: Fig. S1). The Dissociated polyA bulks were used as a control, since the polyA tail capture protocol and the ribosomal depletion protocol might cause specific changes in the bulk data that we have not characterized in this work. Therefore, Classic bulks and Dissociated bulks, both with -rRNA protocols, were compared. The residuals were calculated as in previous sections of this paper for Dissociated and Classic (-rRNA) and Dissociated (polyA).

In contrast to our previous experiments, no ground truth was available for the dataset of real bulks, so we could not compare proportions to the residual matrix’s factors using the previous strategy. Instead, we compared the residual values for each bulk type and used a non-parametric Wilcoxon t-test to compare the distributions.

We also performed factorization of the combined Classic and Dissociated (both -rRNA protocols) residuals with PCA and NMF to evaluate whether the residual would show information of the hypothesized missing adipocytes in a PCA or NMF component. The PCA was implemented using the PCA function from the Scikit-learn library (v.1.4.2) [34]. Scatter plots were generated to visualize the distribution of the two sample types across the principal components. The plots were colored by sample type to facilitate visual differentiation. A two-sample t-test was conducted to identify significant differences between the Classic and Dissociated bulks across the principal components. The analysis was performed for each principal component separately, and p-values were adjusted using the Bonferroni correction to control for multiple comparisons.

For each of the eight principal components, genes were ranked based on their loadings, calculated as the product of the PCA components and the square root of the explained variance. The genes contributing the most to each component were identified and listed in order. This gene list was later used for further biological interpretation by conducting gene ontology analysis using GOrilla [35], a tool for discovering enriched GO terms in ranked lists of genes. We ran a single ranked list of gene modes and searched for gene ontology biological processes. The genes from each principal component were input as a single ranked list, focusing on biological processes. The top 30 significant pathways, based on the lowest p-values, were extracted and plotted to illustrate the variation most associated with the genes contributing to the principal components. The significance of the pathways was visualized through bar plots, displaying the negative logarithm of the *p*-value for each pathway associated with the principal components.

Similarly, NMF was applied to the same residual data with the same software and parameters outlined in the section on residual factorization and evaluation above. A two-sample *t*-test was conducted for each principal and NMF component to identify significant differences between the classic and dissociated bulks. The analysis was performed for each component separately, and *p*-values were adjusted using the Bonferroni correction to control for multiple comparisons. Gene ontology analysis was also performed using GOrilla, and the top 30 significant pathways, based on the lowest *p*-values, were extracted, and plotted to illustrate the processes most associated with the genes contributing to the NMF factors.

## Supplementary Information

Additional file 1: Supplemental Figures (Fig. S) 1-15.

Additional file 2: Supplemental Table 1. (Data origin, links and processing details).

## Data Availability

The data that support the findings of this study are all freely available. The count data can be downloaded through the Gene Expression Omnibus with accession numbers GSE176067 (adipose single-cell), GSE176171 (adipose single-nucleus), adipose bulks (GSE174475) [28], and GSE217517 for HGSOC data [17]. The PBMC data provided by 10x Genomics can be directly accessed through their website [24]. The code developed for this study is available at our GitHub repository [36]: https://github.com/greenelab/pred_missing_celltypes under BSD 3-Clause License, and Zenodo [37]. The repository includes all necessary scripts and a README file with setup instructions and usage guidelines. For updates or assistance, users can refer to the repository or open an issue for queries. Our aim is to support transparency and reproducibility in computational research through this open-access resource.
