# Supplementary material for: Missing cell types in single-cell references impact deconvolution of bulk data but are detectable
Source: Genome Biol. 2025 Apr 7;26:86. doi: 10.1186/s13059-025-03506-9 (PMC11974051; doi:10.1186/s13059-025-03506-9)
Supplement: Supplementary file 1 — Additional file 1: Supplemental Figures (Fig. S) 1-15. [file 13059_2025_3506_MOESM1_ESM.docx]

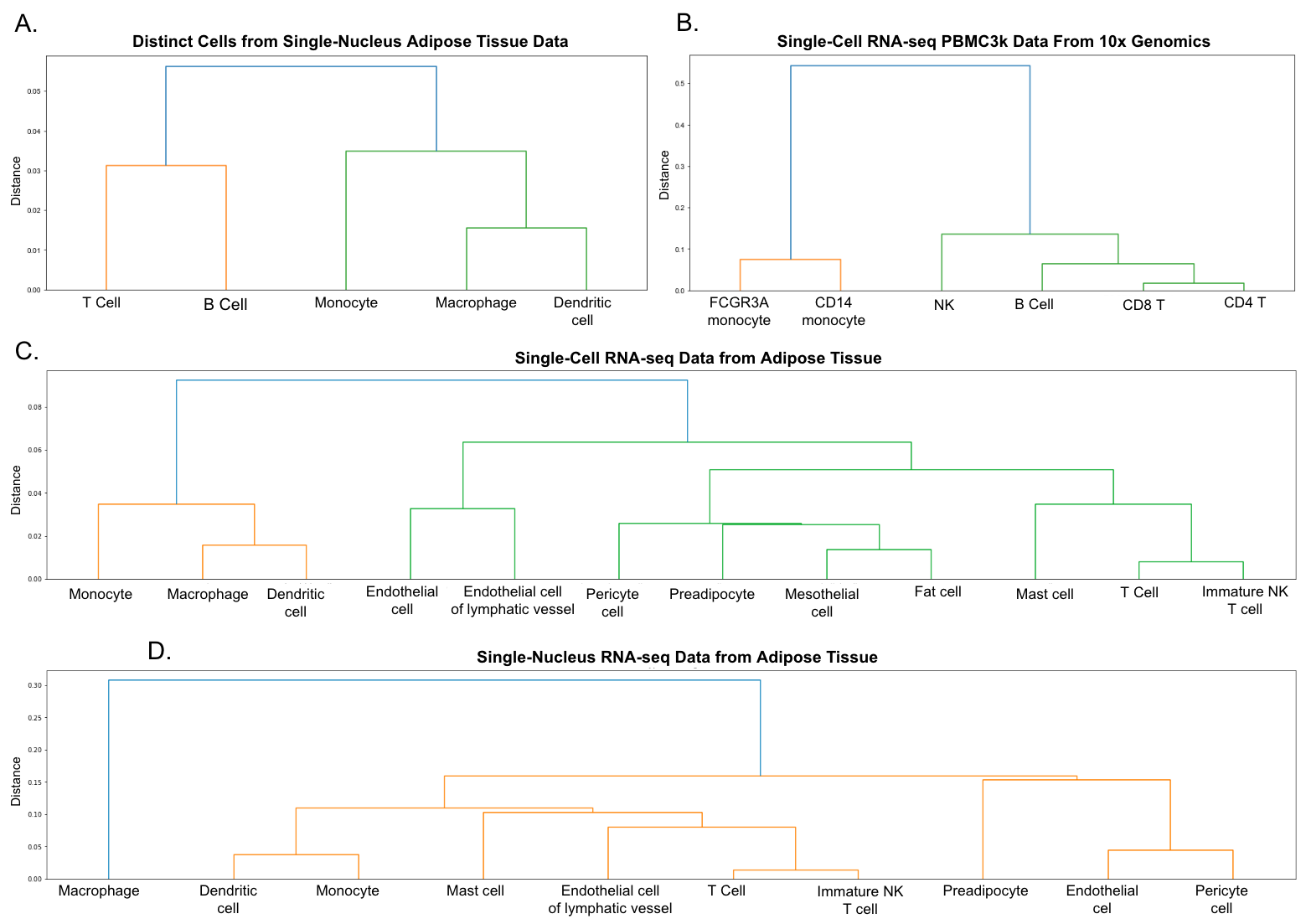


**Fig. S1. Correlation distance dendrogram between all cell types in each of the datasets used, representing differences in gene expression by** **cell type**. Distance is calculated as the inverse of Pearson’s correlation (1 – Pearson’s r) with each cell-type’s expression. The greater the distance, the more different two cell types are. **A.** Dendrogram of 5 curated cell types extracted from single-nucleus adipose tissue dataset (shown in C). **B.** Dendrogram of all cell types extracted from PBMC3k 10x Genomics dataset.


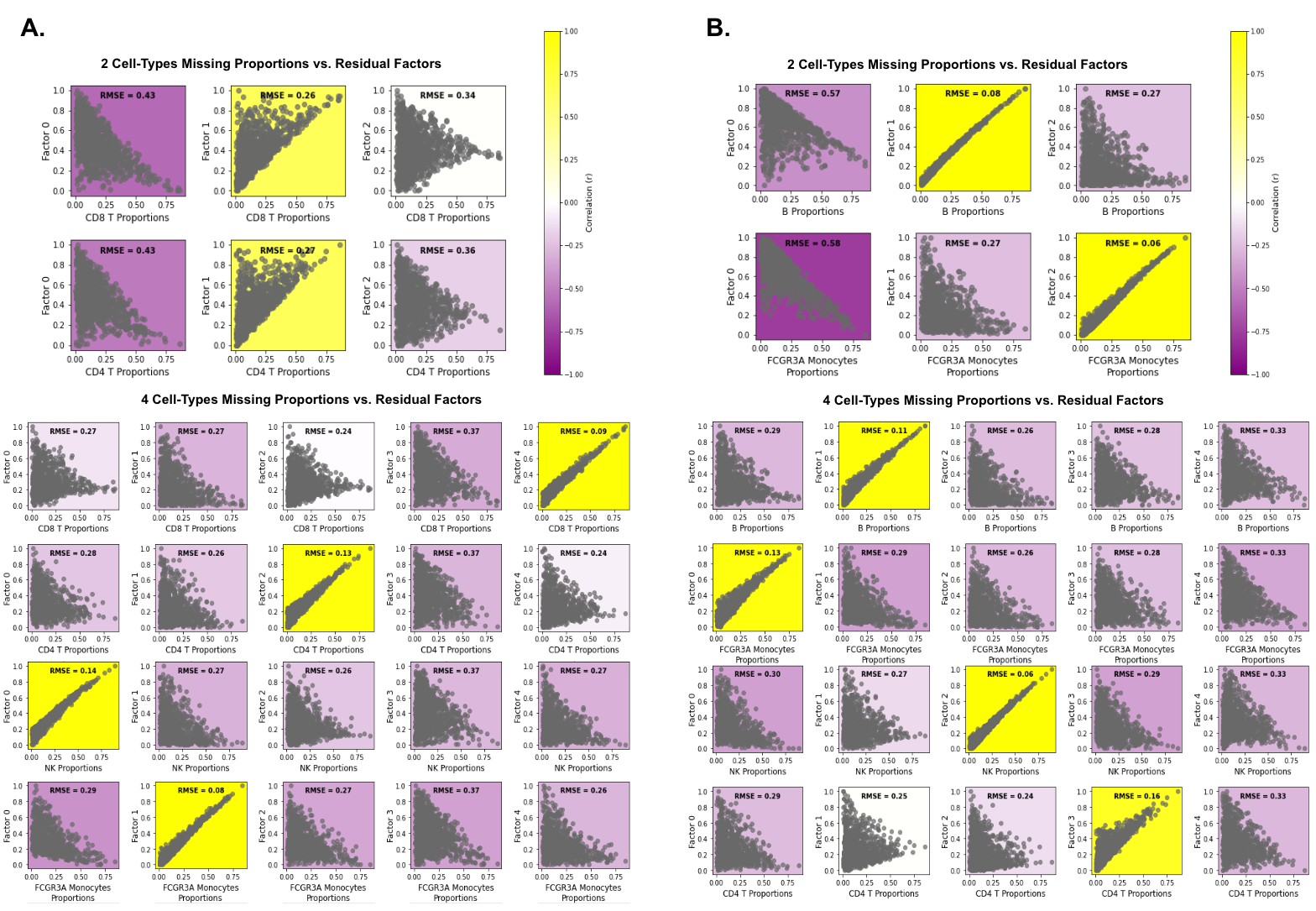


**Fig. S2*.*** ***Non-Negative Least Squares (NNLS) Deconvolution of PBMC3k Pseudobulks with Random Proportions****: A portion of these data is shown in Figure 3 on the main paper.* *We remove one, two, three and four cell types from the deconvolution reference. These cell types are selected to have low correlation in gene expression* ***A.*** *or selected randomly from cells with* *similar expression.* ***B.*** *The residual matrix is calculated and factorized with Non-negative Matrix Factorization (NMF). Each factor is then correlated to each of the missing cell-type’s proportions. Pearson’s correlation (r) (color bar) is shown in the coloring of each plot, and the Root Mean Square Error (RMSE) value between the residual factor and the cell-type proportions are noted. Note: The rest of the data (for one and 3 missing cell types) is in Figure 2 of main text.*


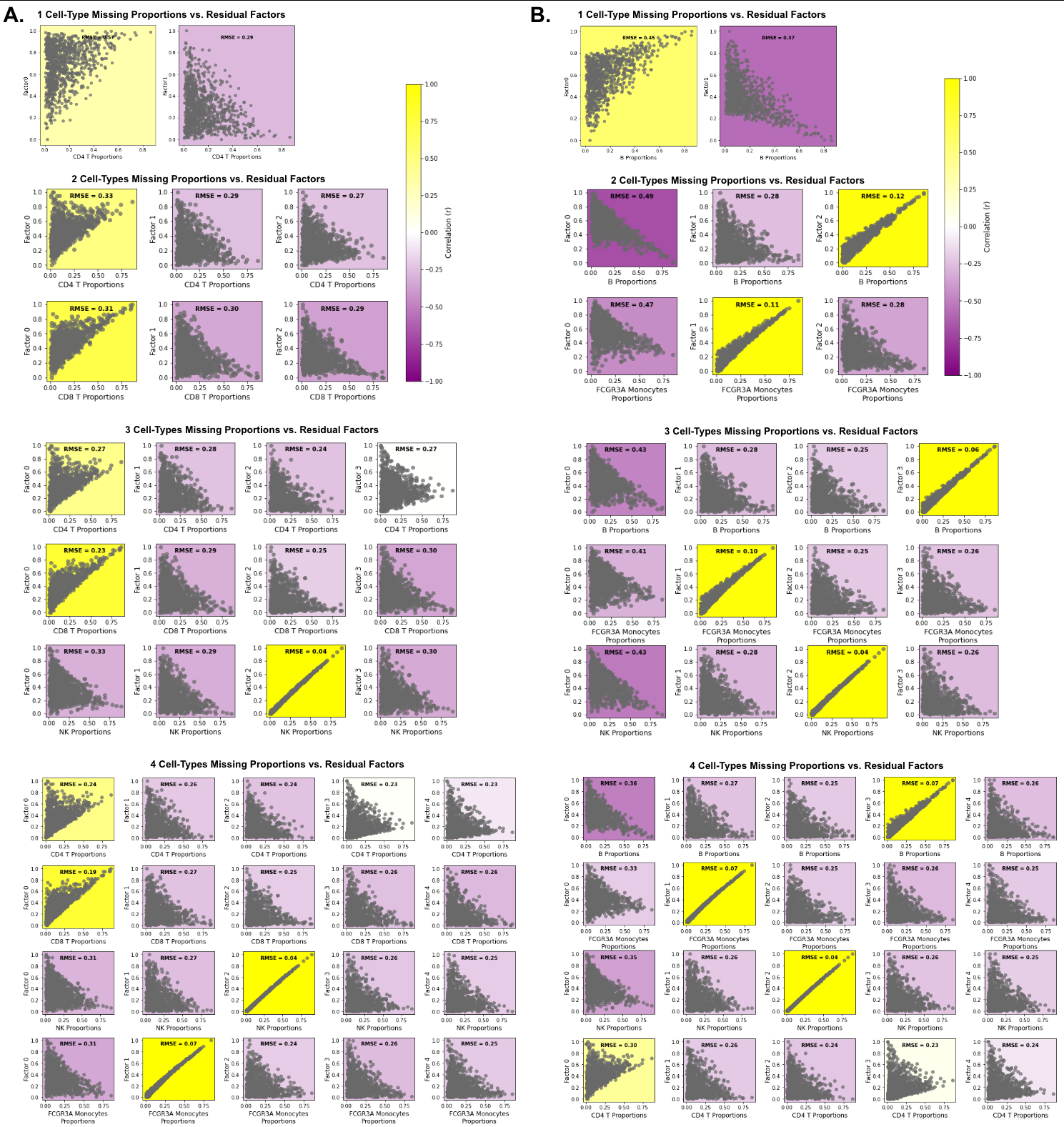


***Fig. S3. BayesPrism Deconvolution of PBMC3k Pseudobulks with Random Proportions.*** *We remove 1, 2, 3 and 4 cell types from the deconvolution reference. These cell types are selected to have low correlation in gene expression.* ***A.*** *or randomly selected cells having similar expression.* ***B.*** *The residual matrix is calculated and factorized with Non-negative Matrix Factorization (NMF). Each factor is then correlated to each of the missing cell-type’s proportions. Pearson’s correlation (r) (color bar) is shown in the coloring of each plot, and the Root Mean Square Error (RMSE) value between the residual factor and the cell-type proportions are noted.*


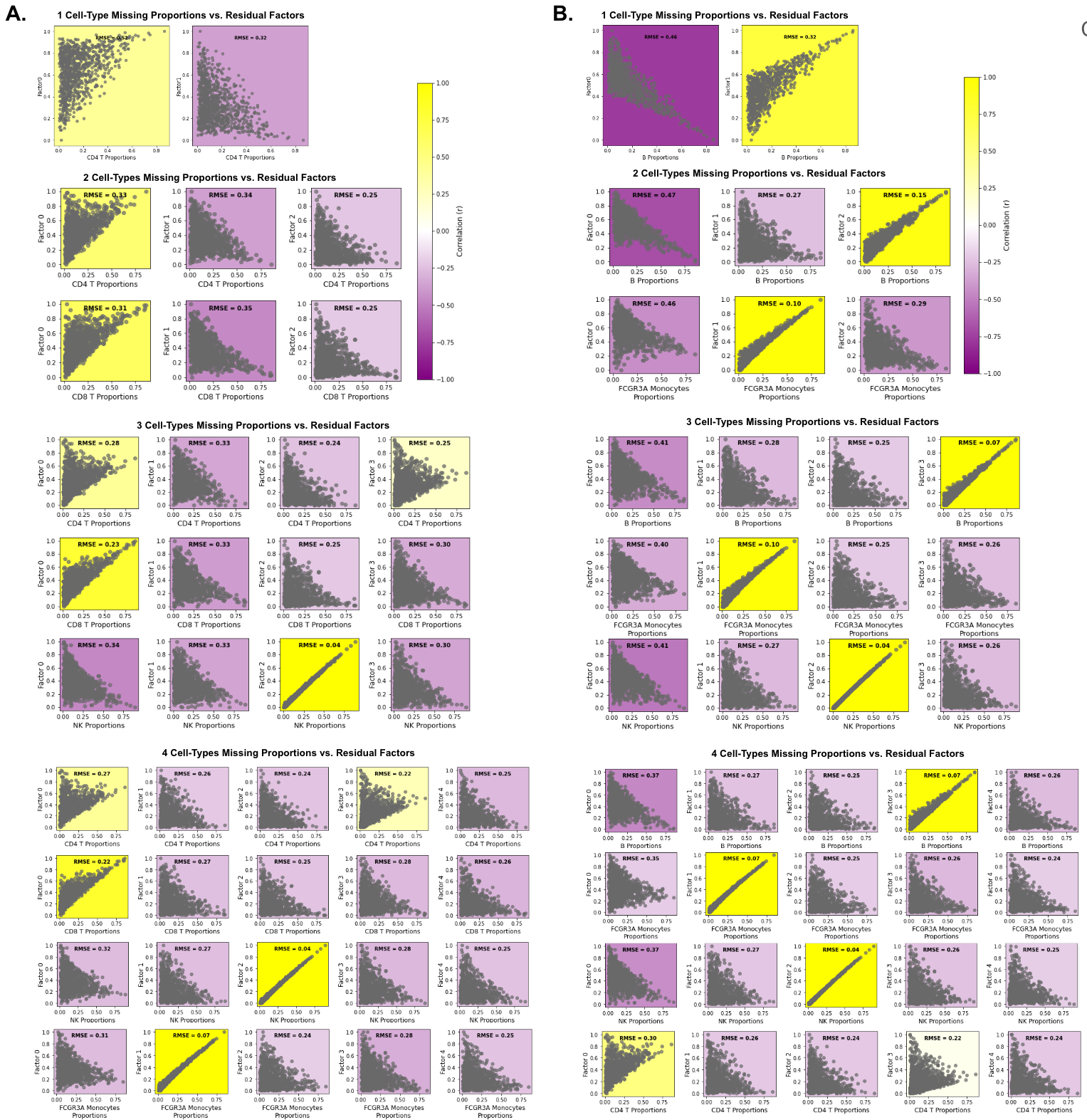


***Fig. S4. CIBERSORTx Deconvolution of PBMC3k Pseudobulks with Random Proportions:*** *We remove 1, 2, 3 and 4 cell types from the deconvolution reference. These cell types are selected to have low correlation in gene expression.* ***A.*** *or randomly selected cells having similar expression.* ***B.*** *The residual matrix is calculated and factorized with Non-negative Matrix Factorization (NMF). Each factor is then correlated to each of the missing cell-type’s proportions. Pearson’s correlation (r) (color bar) is shown in the coloring of each plot, and the Root Mean Square Error (RMSE) value between the residual factor and the cell-type proportions are noted.*

*
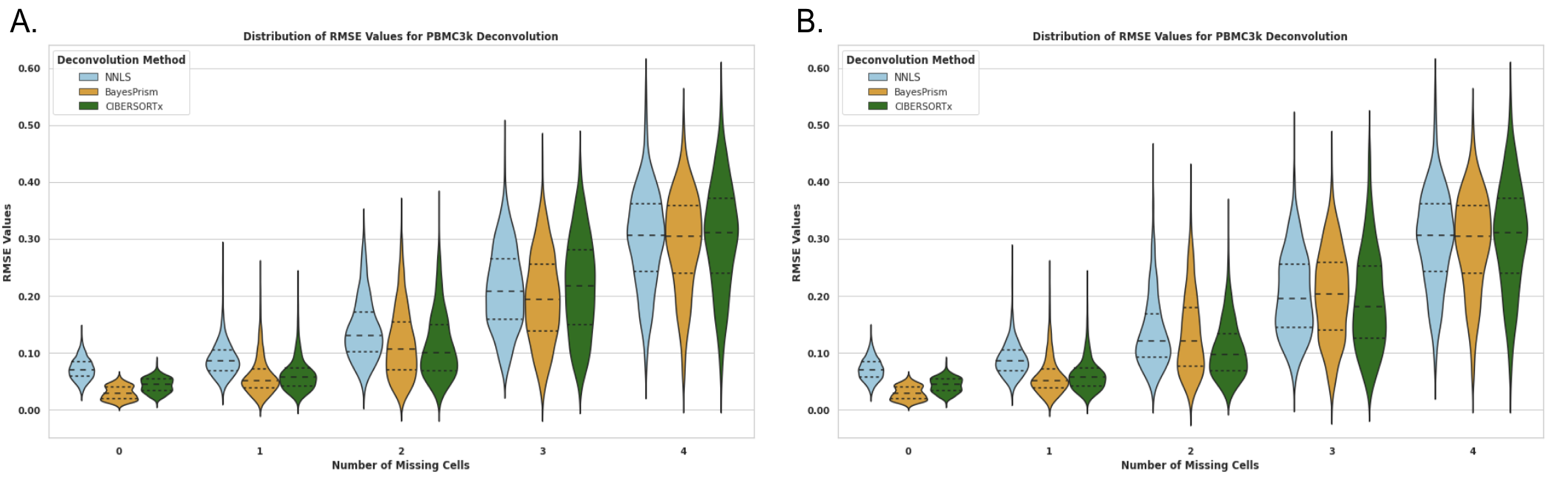
*

***Fig. S5****.* ***Comparison of deconvolution performance in Non-Negative Least Squares (NNLS), BayesPrism and CIBERSORTx for PBMC3k pseudobulks across number of missing cell types. A.*** *Violin plots showing deconvolution performance when deleting cell types that are not highly correlated.* ***B.*** *Violin plots showing deconvolution performance when deleting cell types that are highly correlated. Y axis represents Root Mean Square Error (RMSE) value of calculated proportions vs. real pseudobulk proportions.* *Non-correlated cell types have only slightly better performance than when correlated cell types are removed.*


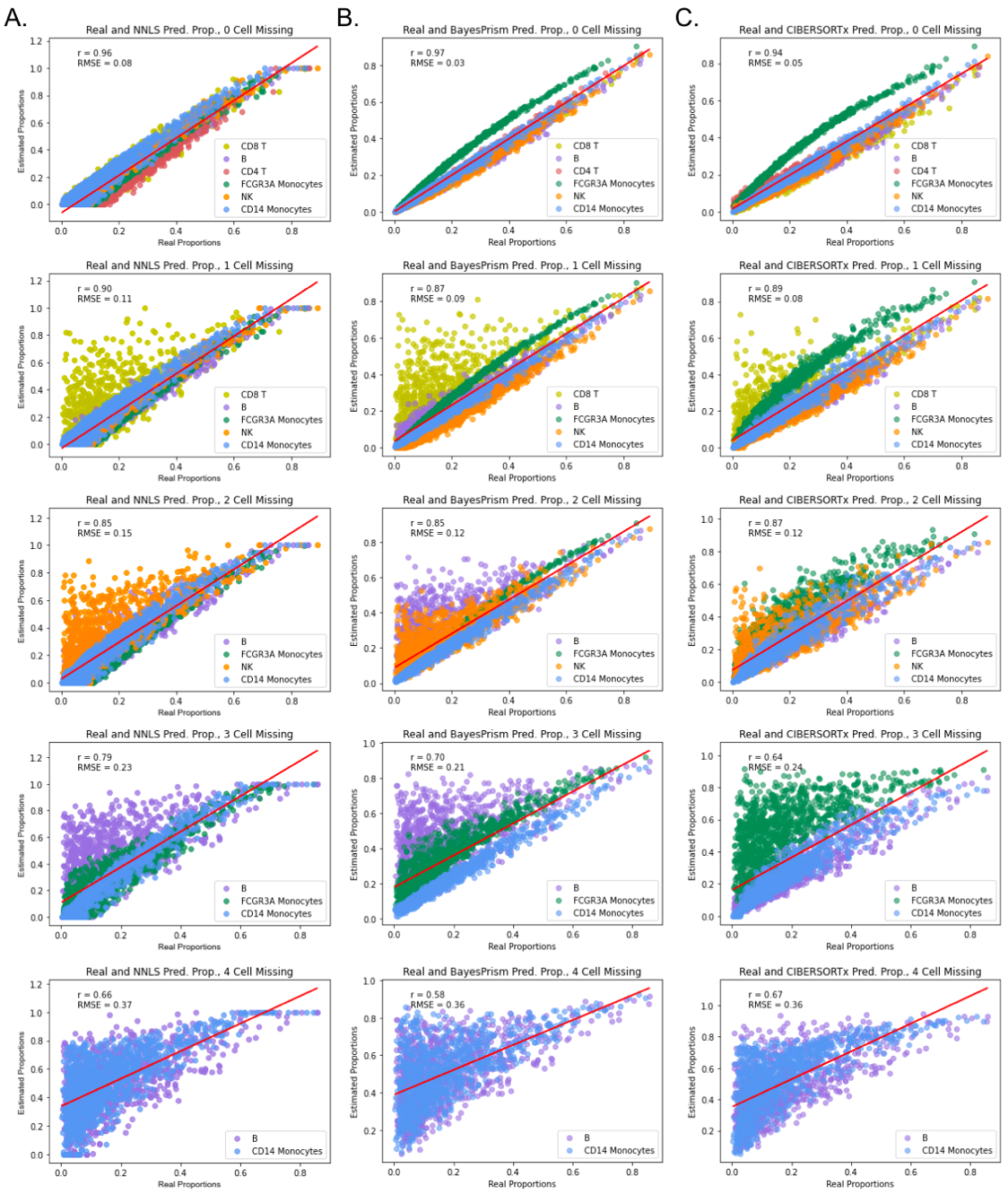


***Fig. S6. Correlated cell types being removed does not impact the performance of the deconvolution methods substantially.*** *From left to right,* ***A.*** *Non-Negative Least Squares (NNLS)****, B.*** *BayesPrism and* ***C.*** *CIBERSORTx performance (real vs. estimated) proportions. First row corresponds to the control (0 missing cell types), second row corresponds to 1 missing cell type, then 2 missing cell types, up to 4 missing cell types in the last row. Pearson’s correlation (r) and Root Mean Square Error (RMSE) values are noted in each plot.*


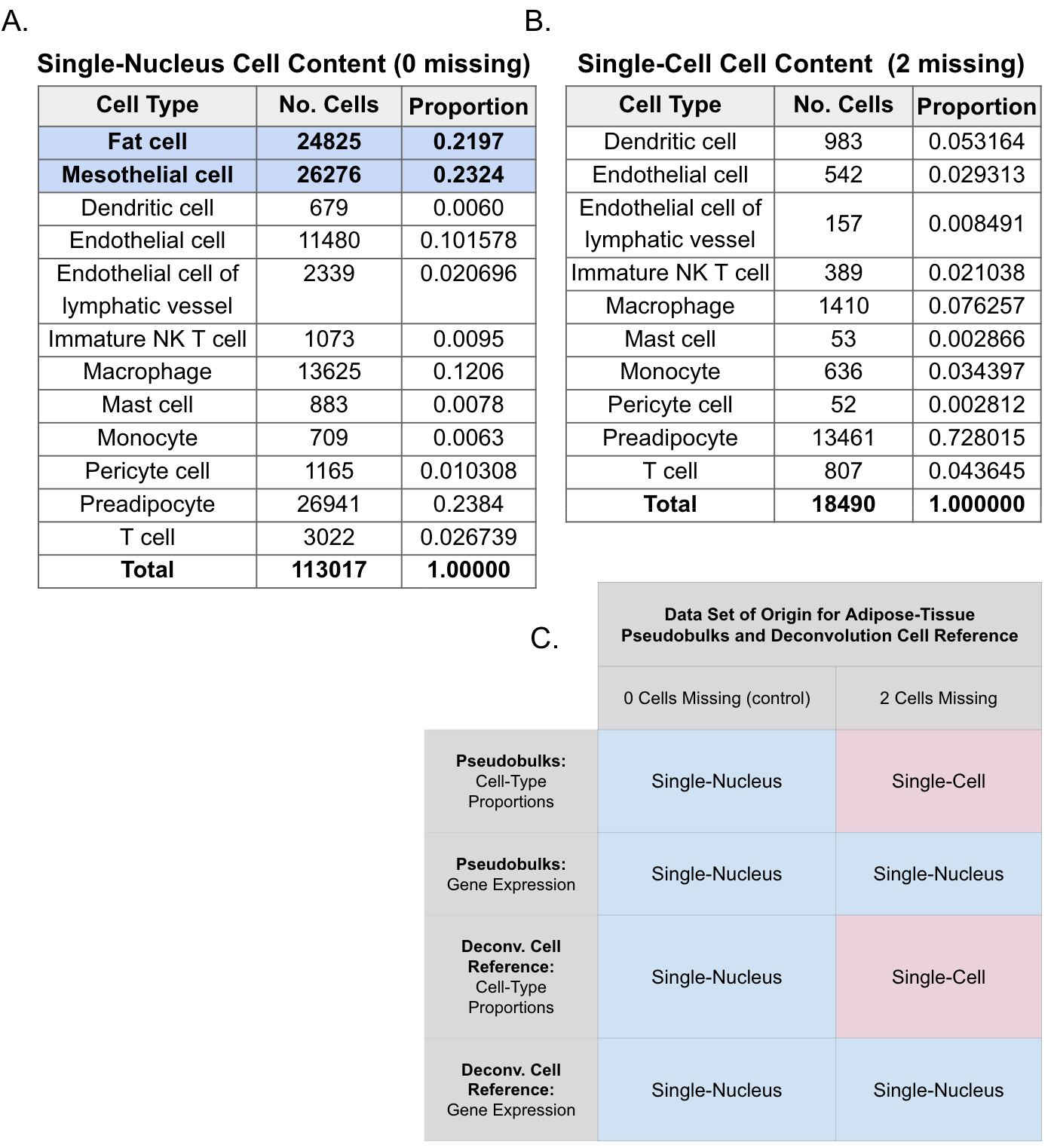


**Fig. S7.** Cell content of both adipose tissue datasets. Single-Nucleus (**A.)** and Single-Cell (**B.).** RNA-seq cell types, number of cells, and proportions. The single-nucleus proportions are used to create the realistic-proportioned pseudobulks, and the single-cell proportions are used to create the cell reference for deconvolution. Cell types that are missing in single-cell are colored in blue. **C.** Pseudobulks created from single-nucleus RNA-seq adipose tissue cell expression, either with random proportions or with single-nucleus proportions (not missing any cell types). Single cell references for deconvolution are either with single-nucleus proportions (no cells missing, control), or using single-cell RNA-seq proportions (two cell types missing).


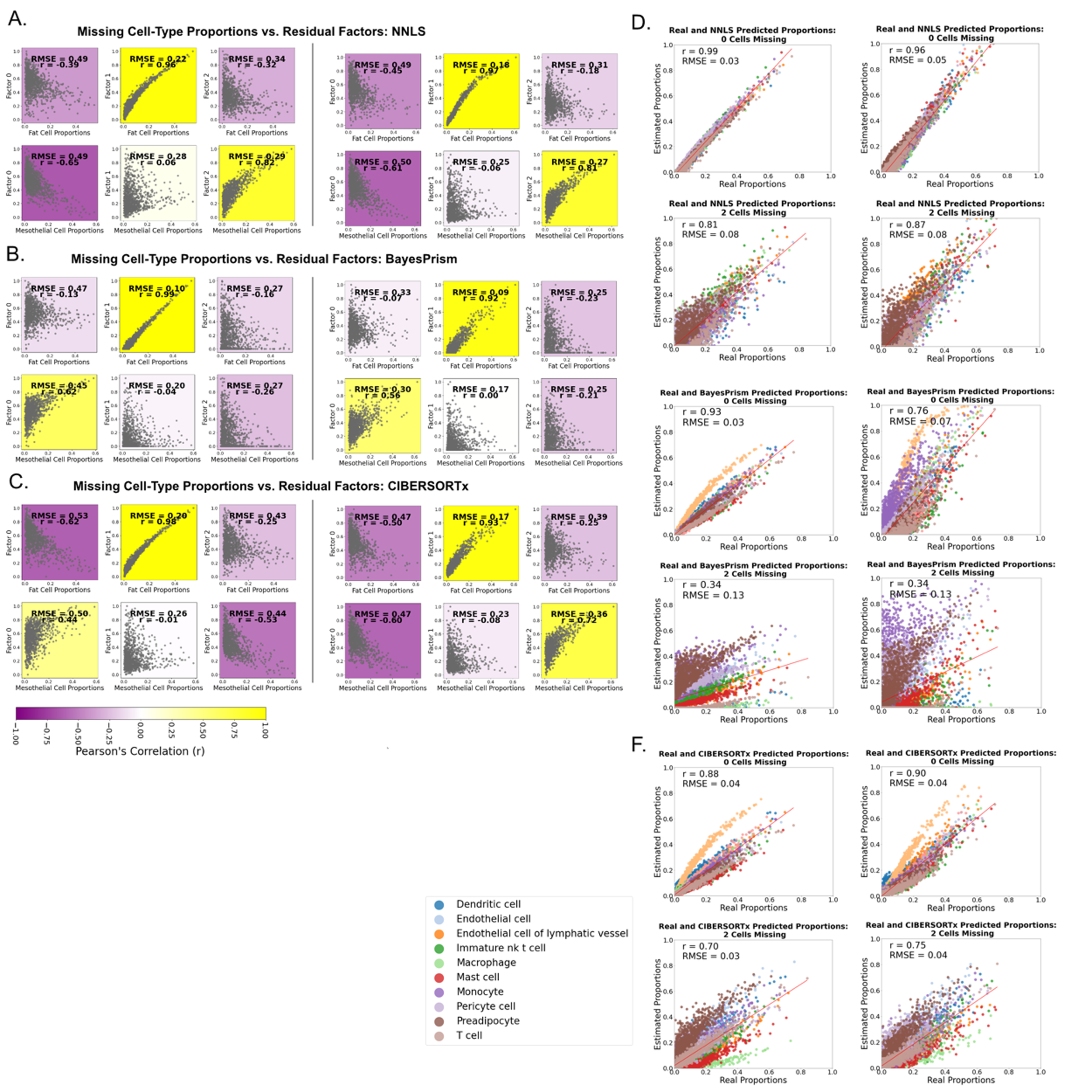


***Fig. S8. Single-cell and Single-nucleus informed pseudobulks, as seen in Figure 4 of the main text, but with random proportions.*** *Panels on the left (A-C) show the residual’s factors of pseudobulks with random proportions compared to missing cell-type’s proportions.* *Plots on the left represent pseudobulks with no noise, and panels on the right represent pseudobulks with noise, each deconvolved with:* ***D.*** *NNLS,* ***E.*** *BayesPrism, and* ***F.*** *CIBERSORTx.* ***The left panel (D-F) shows the real vs. calculated proportions for pseudobulks of realistic proportions in each of the deconvolution methods.*** *The left columns of these panels represent pseudobulks with no noise added, and the right column represents bulks with noise added****. A and D.*** *Non-Negative Least Squares (NNLS) with no noise and NNLS with noise,* ***B and E.*** *BayesPrism with no noise and BayesPrism with noise,* ***C and F.*** *CIBERSORTx with no noise and CIBERSORTx with noise. The top panel of each represents the deconvolution with no cells missing (same cells as present in pseudobulks), and the bottom plot represents the proportions with 2 cells missing (no adipocytes or mesothelial cells), as seen in single-cell RNA-seq. The red line in each plot represents the regression fit line. Each plot has Root Mean Square Error (RMSE) and Pearson’s correlation (r) noted.*


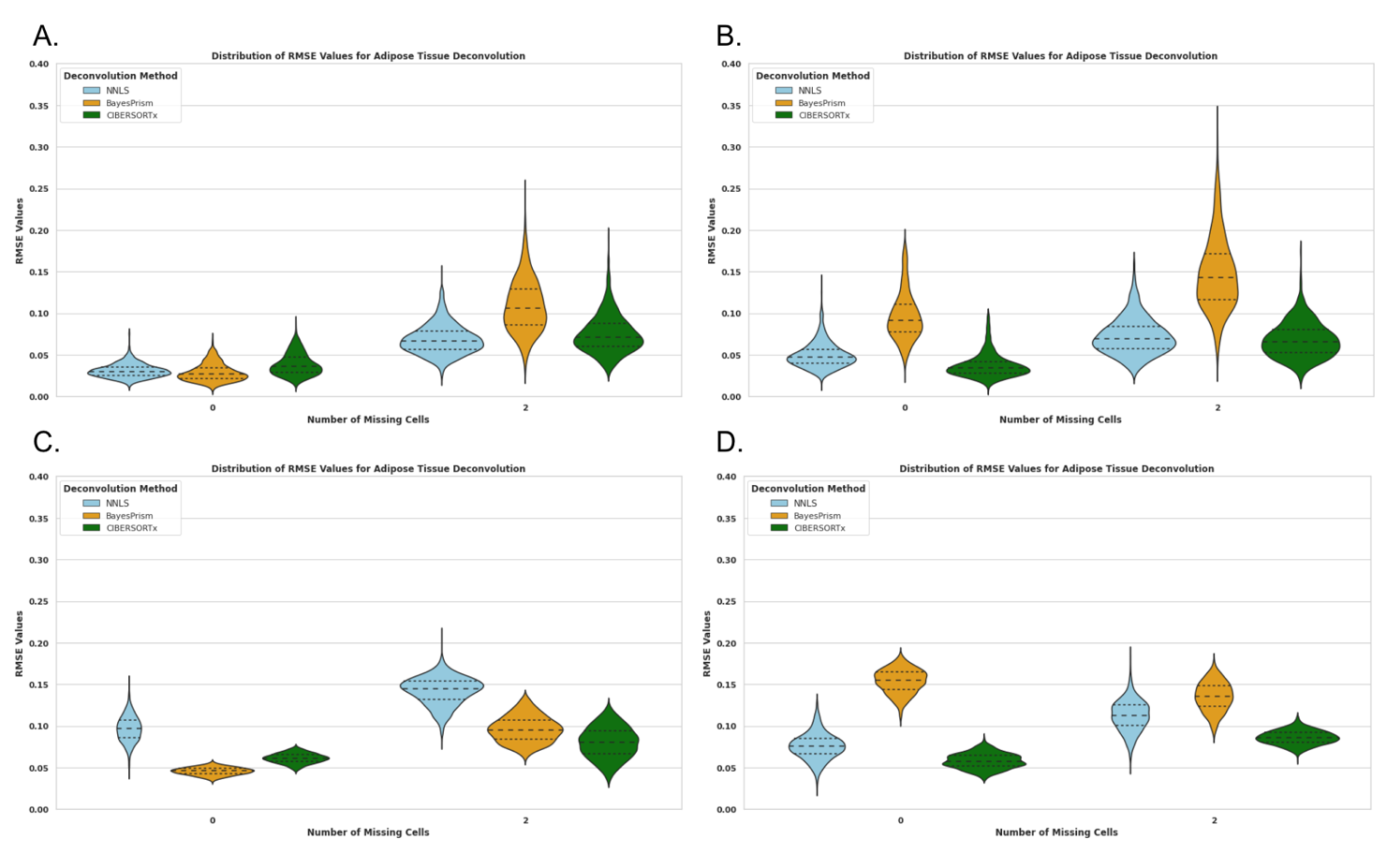


***Fig. S9. Calculated Root Mean Square Error (RMSE) for each deconvolution method****. Panels on the right represent pseudobulks with noise, and panels on the left represent pseudobulks without noise. The top panels represent pseudobulks with random proportions, and the bottom panels represent pseudobulks with realistic proportions.* ***A. No noise, random, B. Noise, random, C. No noise, realistic, D. Noise, realistic.***


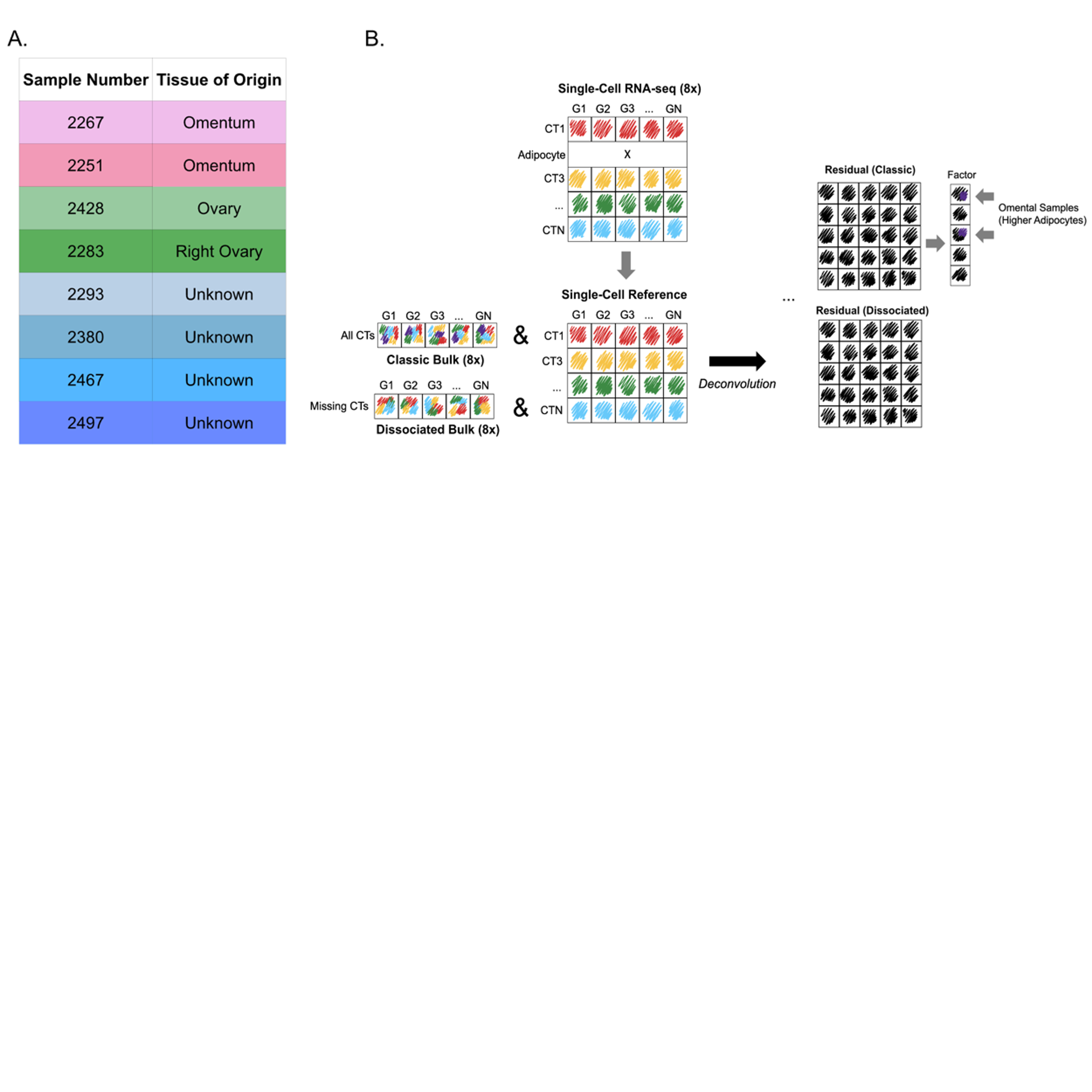


***Fig. S10. Analysis of experimental (not simulated)*** ***classic and dissociated bulks deconvolved with matched single-cell RNA-seq data. A.*** *Table showing the tissue of origin for each sample.* ***B.*** *Schematic illustration of experimental design. The dissociated bulks are hypothesized to match the cell types in the single-cell data, therefore are considered to have no cell types missing. The classic bulks are hypothesized to have at least one cell type missing (adipocytes). The residual is calculated as previously, and one of the residual’s factors is expected to match adipocyte proportions.*


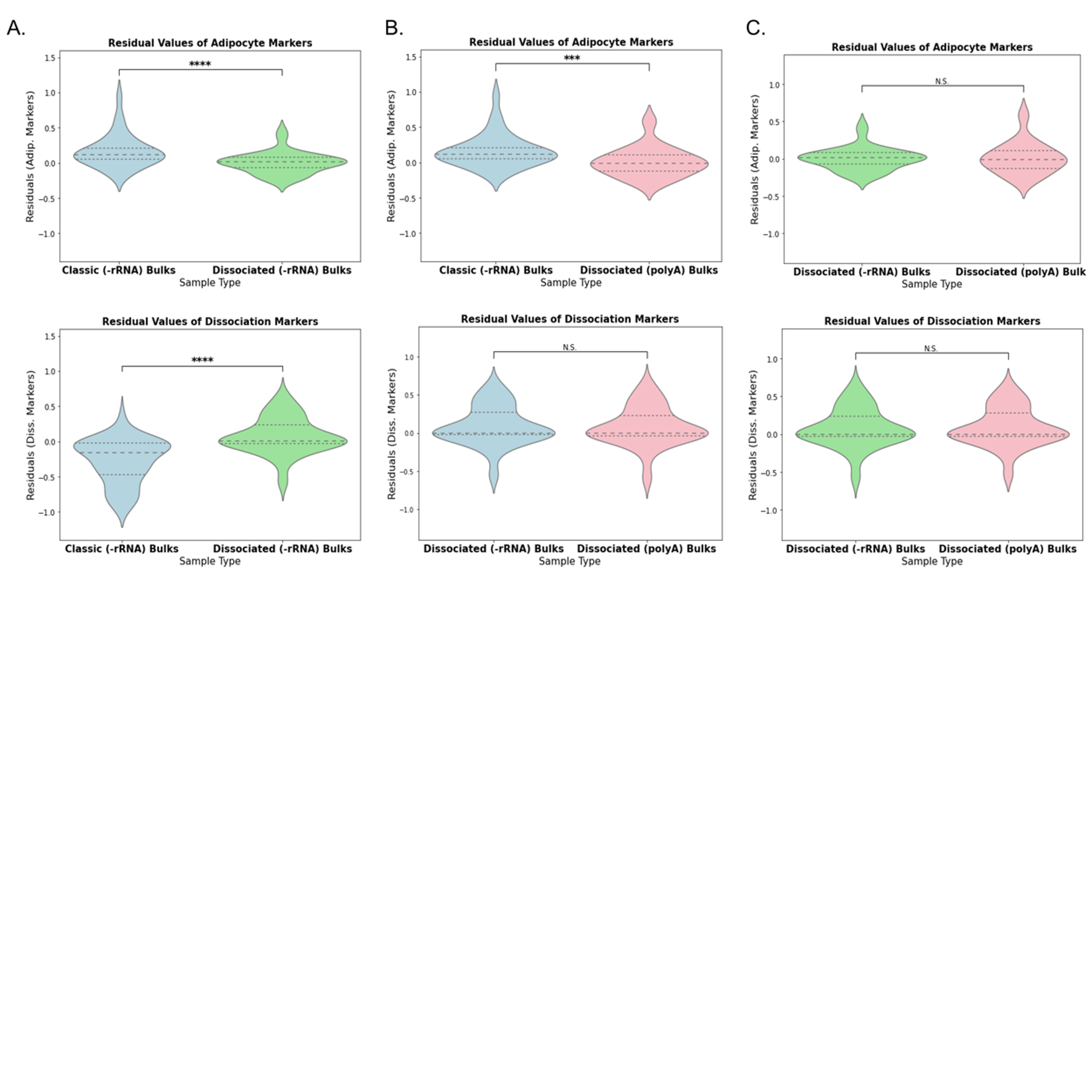


***Fig. S11. Violin plots of the Residual values in Adipocyte Markers (top), and Dissociation Response Markers (bottom).*** ***A.*** *Compares the adipocyte and dissociation values in the residuals of Classic and Dissociated Bulks (both -rRNA).* ***B.*** *Compares the adipocyte and dissociation values in the residuals of both Dissociated Bulks (poly-A tail enriched and captured (polyA) and ribosomal RNA depleted (-rRNA)).* ***C.*** *Compares the adipocyte and dissociation values in the residuals of Classic Bulks (-rRNA) and Dissociated Bulks (polyA). Asterisks in all plots mark the statistical significance of p-values computed with Wilcoxon T-Test.*

***Supplemental Figure 10. Violin plots of the Residual values in Adipocyte Markers (top), and Dissociation Response Markers (bottom).*** ***A.*** *Compares the adipocyte and dissociation values in the residuals of Classic and Dissociated Bulks (both -rRNA).* ***B.*** *Compares the adipocyte and dissociation values in the residuals of both Dissociated Bulks (polyA and -rRNA).* ***C.*** *Compares the adipocyte and dissociation values in the residuals of Classic Bulks (-rRNA) and Dissociated Bulks (polyA). Asterisks in all plots mark the statistical significance of p-values computed with Wilcoxon T-Test.*


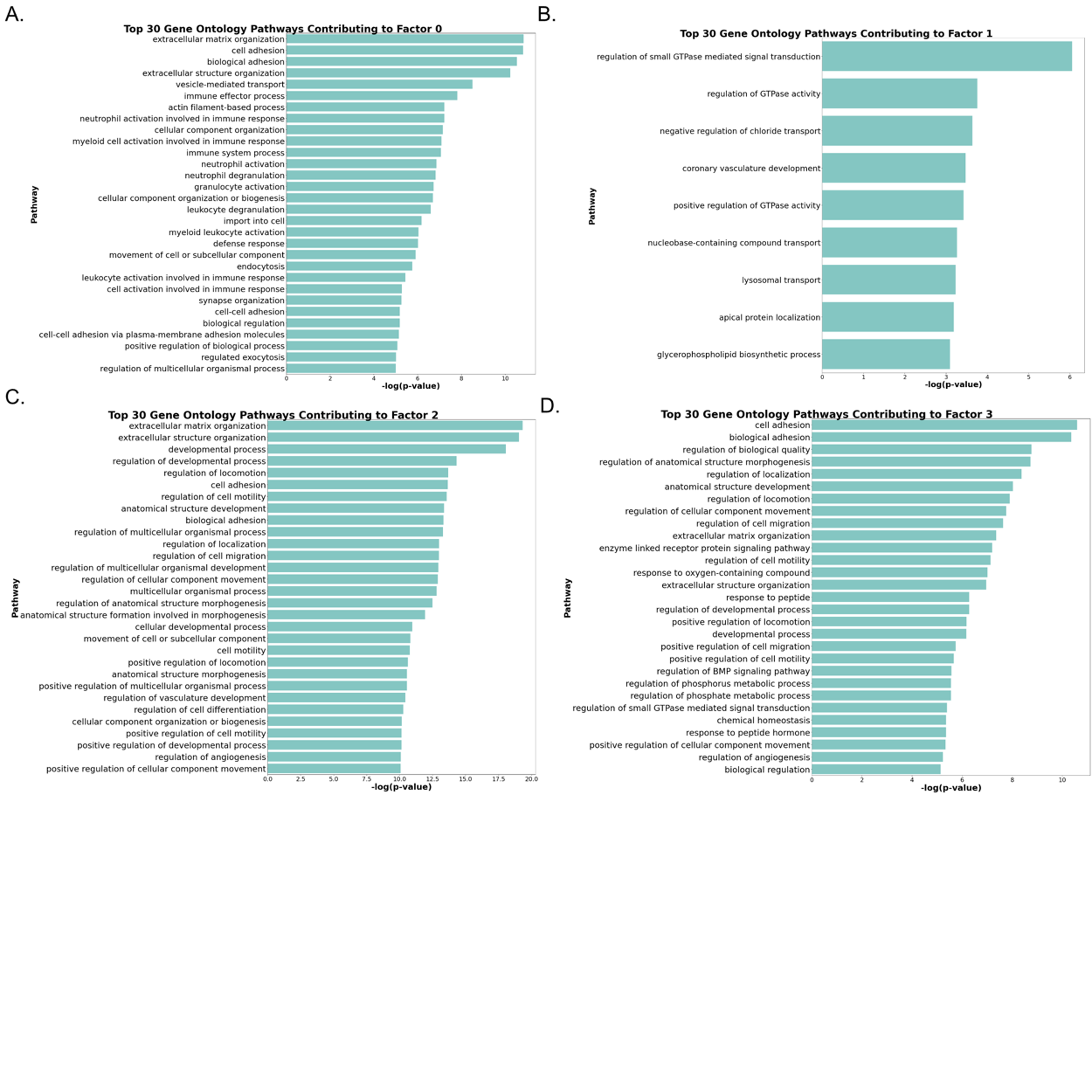


**Fig. S12. Non-negative Matrix Factorization (NMF) Analysis and Associated Gene Ontology Pathways of High Grade Serous Ovarian Cancer (HGSOC) Dissociated and Classic bulks. A-D:** Bar plots of the top 30 Gene Ontology (GO) pathways for each of the four NMF factors (0-3). Pathways are ranked by the p-values, and significance is indicated on a logarithmic scale.


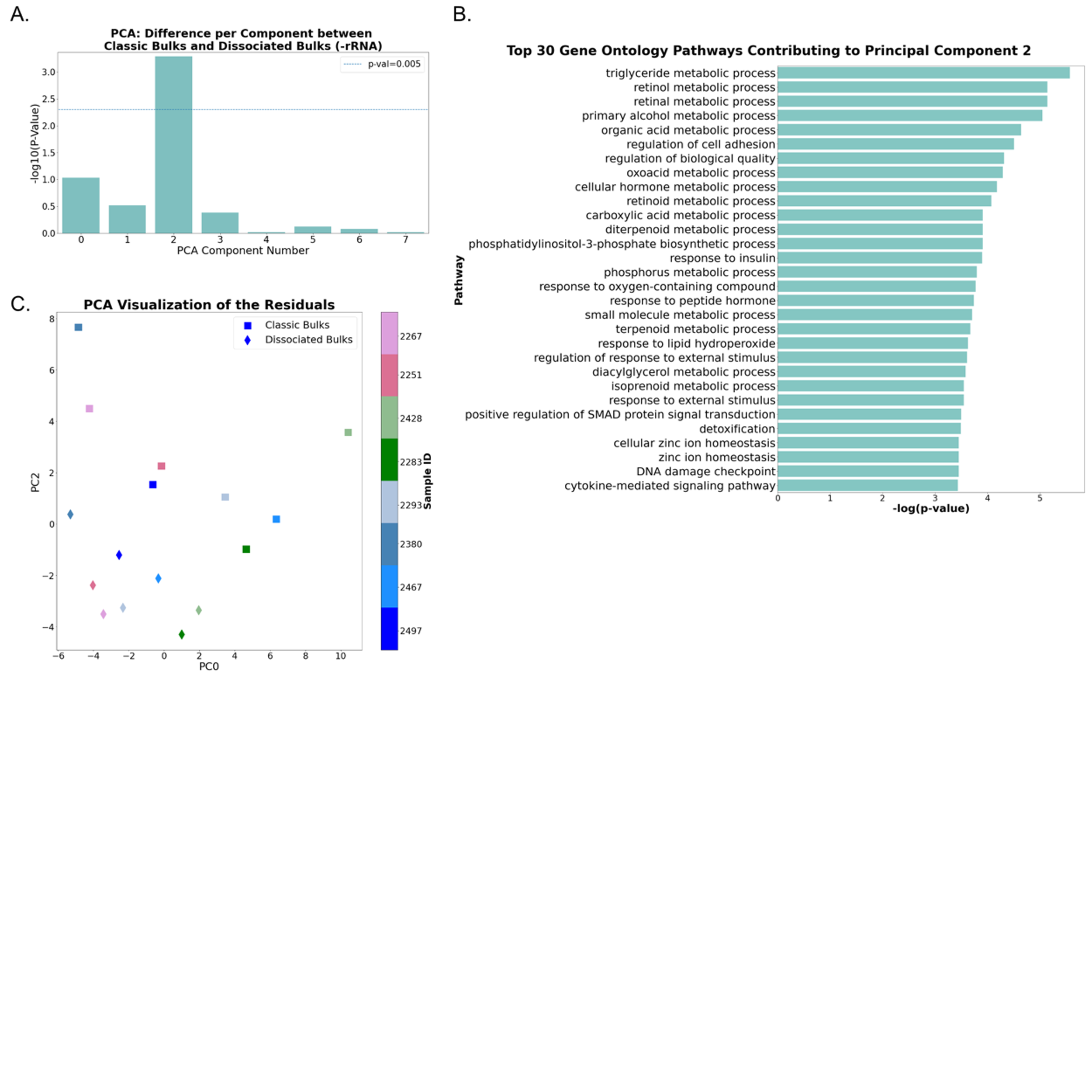


**Fig. S13. Factorization and Visualization of the Residuals of Classic Bulks Dissociated bulks (both ribosomal RNA depleted (-rRNA)) of High Grade Serous Ovarian Cancer (HGSOC) combined. A.** PCA (Principal Component Analysis) of each sample, classic and dissociated, within the PC0 and PC2 space. **B. Paired t-test results showing the log(p-values) for each of t**he means of the principal components. While most components showed no significant differences (Components 0, 1, 3-7; all p-values > 0.05), Component 2 demonstrated a statistically significant difference with a t-statistic of 4.41 and a p-value of 0.000595. **C.** Gene pathways from gene ontology analysis using GOrilla on the ordered genes contributing to Principal Component 2.


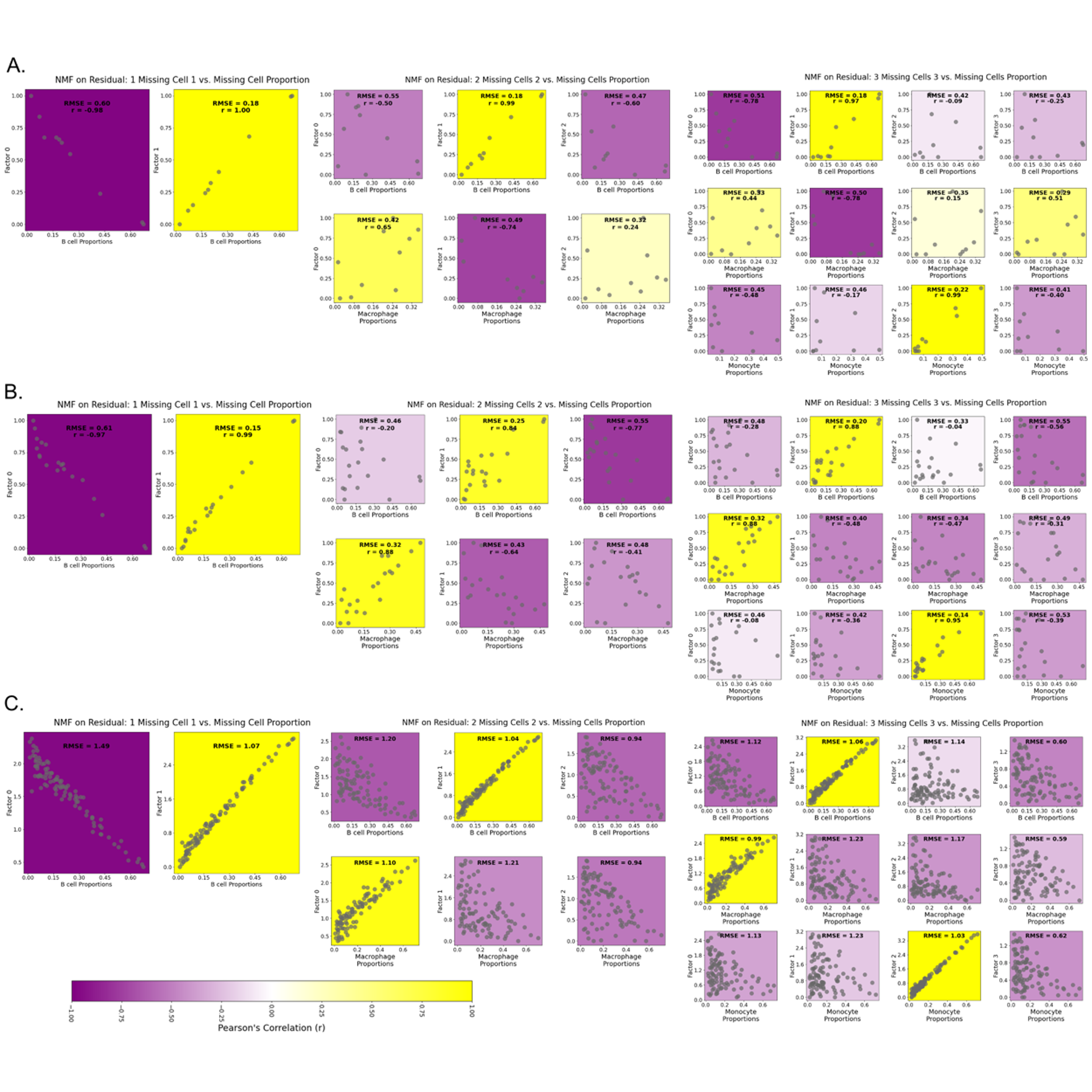


**Fig. S14. Recovery of Missing Cell Types Through Residual Factorization.** The figure illustrates the methodology of recovering missing immune cell types using residual factorization with varying numbers of bulk samples, from top to bottom, **with A.** 10, B. 20, and **C.** 100. Each panel shows the recovery pattern when tested on the same dataset, consisting of 5 distinct immune cell types. The recovery was performed with 1, 2, and 3 cell types removed from the reference (left to right). Despite the varying number of bulks used, the recovered patterns across all panels are highly consistent, demonstrating the robustness of the residual factorization method in recovering missing cell types across different conditions.


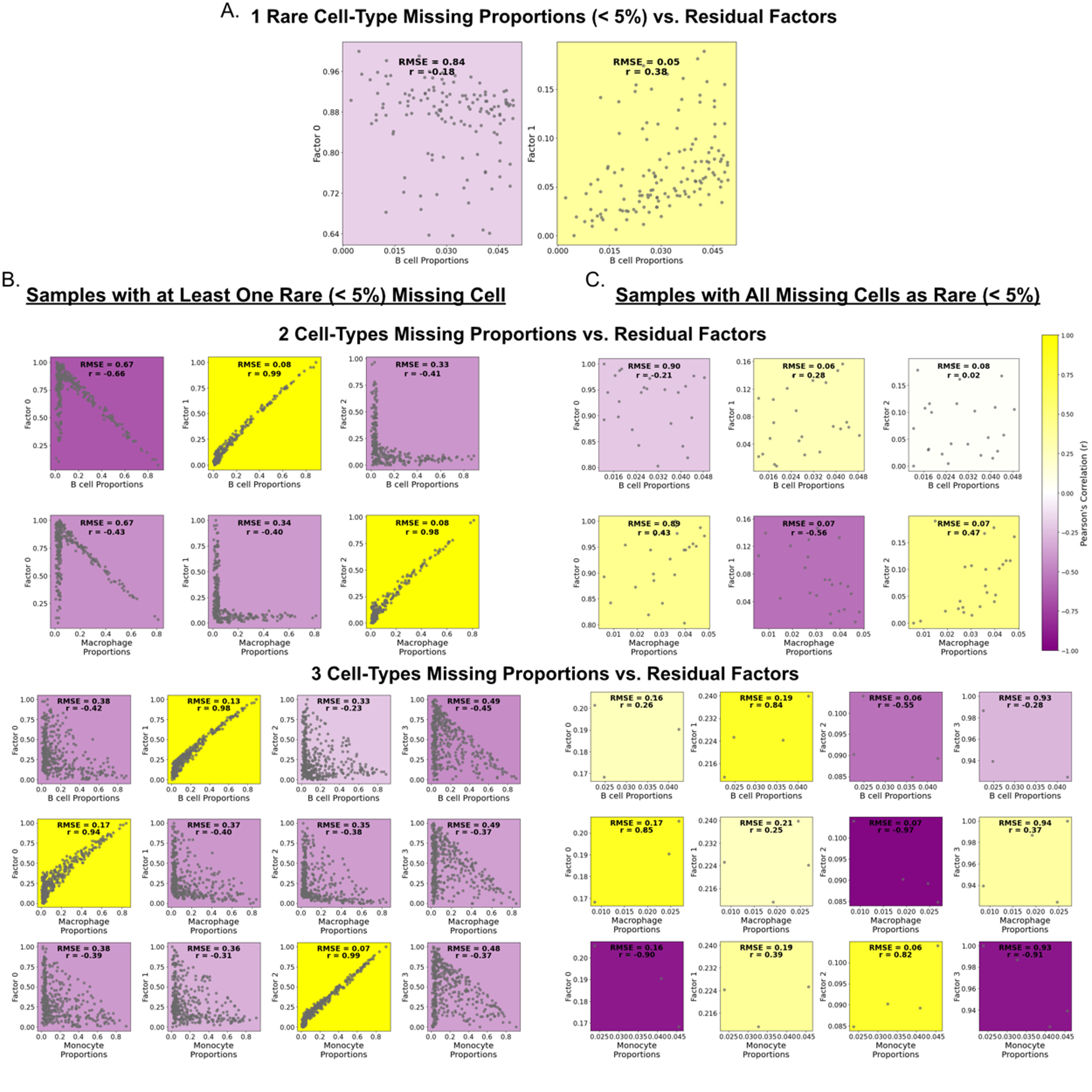


**Fig. S15. Recovery of Missing Cell Types Through Residual Factorization of Rare Cell Types (less than 5%).**
The figure illustrates the methodology of recovering missing immune cell types using residual factorization when the missing cell is in low proportions, less than 0.05. This analysis uses the same data as Figure 1 in the main paper. A. One missing cell type, B cells, are missing from reference but only present in less than 0.005 proportions in samples. Panel B shows results at least one of the missing cell types is rare (< 5% of sample). Panel C shows shows results all missing cell types are rare.
